# A polydopamine nanomedicine used in photothermal therapy for liver cancer knocks down the anti-cancer target NEDD8-E3 ligase ROC1 (RBX1)

**DOI:** 10.1186/s12951-021-01063-4

**Published:** 2021-10-15

**Authors:** Zhanxia Zhang, Junqian Zhang, Jianhui Tian, Hegen Li

**Affiliations:** 1 Cancer Institute, Longhua Hospital, Shanghai University of Traditional Chinese Medicine, 725 Wanping South Road, Shanghai, 200032 China; 2 Department of Medical Oncology, Longhua Hospital, Shanghai University of Traditional Chinese Medicine, 725 Wanping South Road, Shanghai, 200032 China

**Keywords:** ROC1, Neddylation, siRNA-loaded nanomedicine, Photothermal therapy, Targeted delivery

## Abstract

Knocking down the oncogene ROC1 with siRNA inhibits the proliferation of cancer cells by suppressing the Neddylation pathway. However, methods for delivering siRNA in vivo to induce this high anticancer activity with low potential side effects are urgently needed. Herein, a folic acid (FA)-modified polydopamine (PDA) nanomedicine used in photothermal therapy was designed for siRNA delivery. The designed nanovector can undergo photothermal conversion with good biocompatibility. Importantly, this genetic nanomedicine was selectively delivered to liver cancer cells by FA through receptor-mediated endocytosis. Subsequently, the siRNA cargo was released from the PDA nanomedicine into the tumor microenvironment by controlled release triggered by pH. More importantly, the genetic nanomedicine not only inhibited liver cancer cell proliferation but also promoted liver cell apoptosis by slowing ROC1 activity, suppressing the Neddylation pathway, enabling the accumulation of apototic factor ATF4 and DNA damage factor P-H2AX. Combined with photothermal therapy, this genetic nanomedicine showed superior inhibition of the growth of liver cancer in vitro and in vivo. Taken together, the results indicate that this biodegradable nanomedicine exhibits good target recognition, an effective pH response, application potential for genetic therapy, photothermal imaging and treatment of liver cancer. Therefore, this work contributes to the design of a multifunctional nanoplatform that combines genetic therapy and photothermal therapy for the treatment of liver cancer.

**Supplementary Information:**

The online version contains supplementary material available at 10.1186/s12951-021-01063-4.

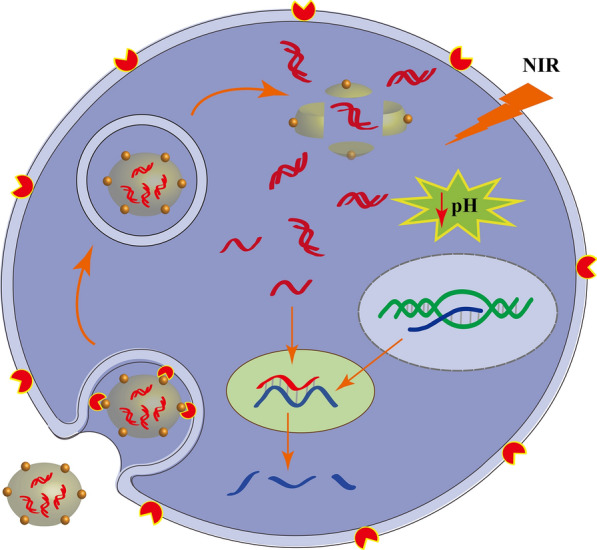

## Introduction

Liver cancer is one of the most common human malignant tumors with high morbidity and extremely high mortality rates [1]. Liver cancer not only seriously endangers human health and life safety, traditional anticancer drugs are characterized by low efficiency and highly toxic side effects, which severely restrict the therapeutic efficiency of liver cancer [2]. Therefore, people urgently hope to find highly efficient and selective anticancer drugs that induce little side effects.

**Fig. 1:**
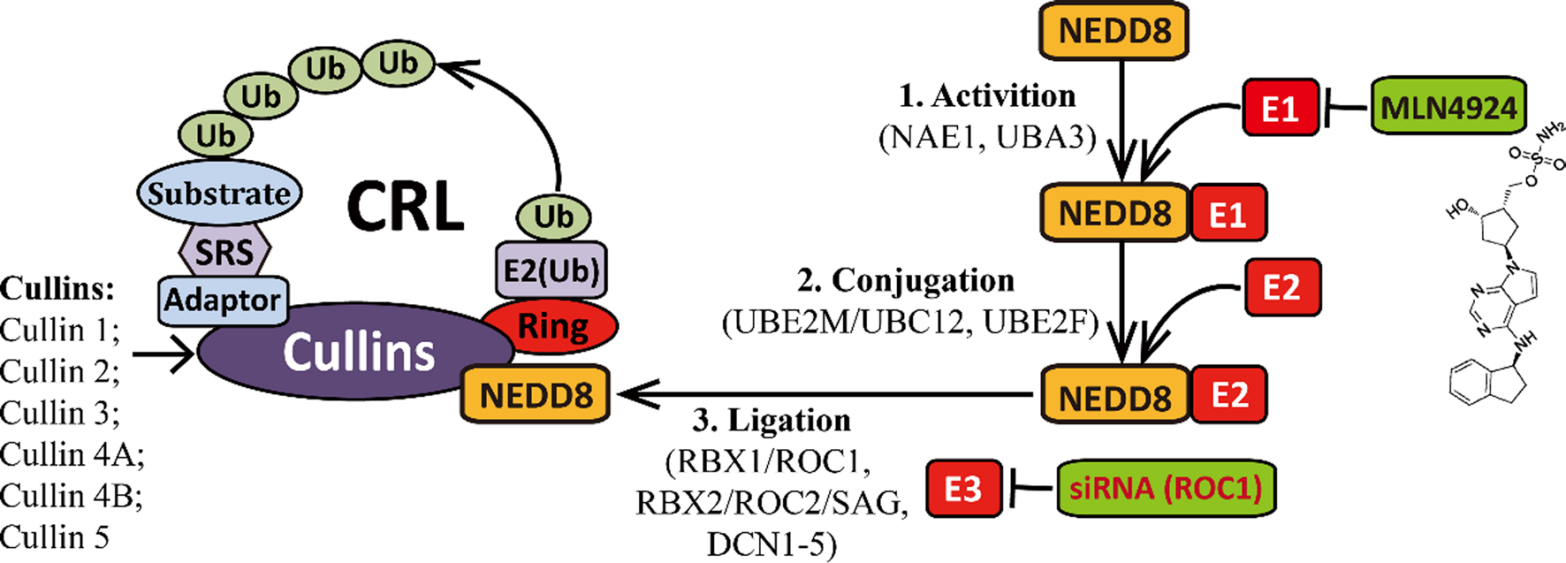
Neddylation modification and its major anticancer targets

Neddylation is a newly discovered posttranslational protein modification pathway that regulates the biological activity of target proteins by binding and degrading them [3]. As shown in Fig. 1, Neddylation modification is an energy-consuming cascade of reaction processes [4]. First, the ubiquitin-like small molecule NEDD8 (neural precursor cell-expressed developmentally downregulated 8) is activated by an E1 NEDD8-activating enzyme (NAE, a heterodimer consisting of NAE1 and UBA3) in the presence of ATP [5]. Then, activated NEDD8 is transferred to an E2 NEDD8-conjugating enzyme (UBC12/UBE2M or UBE2F) [6]. In the final step, NEDD8 is covalently linked with the target substrate under the action of E3 NEDD8 ligase (ROC1/RBX1, ROC2/RBX2/SAG, DCN1-5, MDM2, etc.) [7]. The substrates of Neddylation are members of the Cullin family (mainly, Cullin-1, -2, -3, -4A, -4B, and -5), which are involved in the assembly of the established Cullin-RING E3 ubiquitin ligases (CRLs) [8]. A CRL consists of a ring structure domain (including ROC1/RBX1 or ROC2/RBX2/SAG), Cullin, a substrate recognition subunit (SRS) and an adaptor [9]. CRLs represent the largest E3 ubiquitin ligase family, which participates in regulating many important biological processes, including the cell cycle, gene transcription, DNA replication and cell apoptosis [10]. Importantly, CRL regulation disorders can lead to various diseases, notably, tumor formation. Highly activated Neddylation and highly expressed NAE1, UBC12 and ROC1 have been detected in the tissues of liver cancer patients [11]. However, when these targets are knocked down, the Neddylation modification is blocked, and the development of liver cancer is inhibited.

RNA interference (RNAi) is a double-stranded RNA-induced homologous sequence-specific posttranscriptional gene silencing process that can specifically degrade the expression of mRNA and inhibit the production of target proteins [12]. siRNA can inhibit the expression of target genes with high efficiency and specificity [13]. However, its application in tumor therapy is limited by its untargeted delivery (off-target effects), insufficient cell and tissue penetration, easy enzymatic hydrolysis and short circulation life in vivo. [14] Currently, there are three main delivery technologies used in the field of gene therapy, namely, physical transfection technology, virus vector technology and nonviral nanocarrier systems [15]. Electroporation is the most commonly used physical delivery method of siRNA and is difficult to perform in vivo [16]. Lentivirus, adenovirus and adeno-associated viruses, as the most widely used viral vectors, have potential safety disadvantages (immunogenicity and mutagenicity) [17]. In contrast, nonviral nanocarriers have favorable immunogenicity and safety [18]. Currently available nonviral nanocarriers mainly include cationic polymers, liposomes, dendrimers, calcium phosphates, etc. [19] Polyethylene imine (PEI) is the gold standard polymer with excellent transfection efficiency, and its derivative (GMP in vivo-jet PEI) has been tested in clinical trials with a variety of diseases [20]. PEI not only protects the stability of genetic drugs but also helps molecular escape from lysosomes through a "proton pump" mechanism to achieve gene transfection in cancer cells [21].

Nanomedicine has been greatly beneficial to cancer treatment due to its advantages of the prolonged elimination half-life of drugs, targeted delivery, controlled release, penetration through body barriers (e.g., blood–brain barrier), etc. [22] Recent studies have shown that targeted molecules-mediated endocytosis of nanomedicine possesses better therapeutic effect and lower side effects after accumulation in tumor tissues via passive EPR (enhanced permeability and retention) effect of tumor [23]. Target molecules normally include small molecules (folic acid, lectin, etc.), polypeptides, polysaccharides, antibodies and nucleic acid aptamers. [24] The folate receptor (FR) is overexpressed in a variety of malignant tumors, and its expression is lower in paracancerous and normal tissue, making it an attractive tumor-specific molecular target [25]. In addition to targeted delivery, the new generation of nanomedicines should be able to release the delivered anticancer drugs in a controlled manner [26]. Researchers have explored a number of intelligent responsive nanomedicines, such as those that respond to external stimuli (light, magnetic fields, ultrasound, etc.) or internal stimuli (pH, temperature, enzymes, redox potential, etc.) [27]. A large number of studies have shown that the pH level of tumor tissues (6.5–6.9) is generally lower than that of paracancerous and normal tissues (7.2–7.4) [28]. This is mainly due to the high-rate aerobic glycolysis (Warburg effect) in tumor tissue and the production of lactates and protons [29]. Based on the low pH of the tumor microenvironment, designed pH-responsive nanomedicines have extensive application prospects.

Photothermal therapy (PTT) is an emerging tumor treatment strategy that leverages hyperthermia generated from absorbed near-infrared (NIR) light energy by photo-absorbing agents to kill tumor cells through thermal ablation and by overcoming the effect of chemotherapy resistance and inhibiting tumor metastasis [30]. Photothermal reagents include mainly organic photosensitive molecules (indocyanine green, methylene blue, etc.) and inorganic materials (precious metal nanoparticles (NPs), metal chalcogenide nanomaterials, carbon nanomaterials, quantum dots, etc.) [31]. However, organic photosensitive molecules have a short half-life in blood and are not selectively enriched in tumor areas, and inorganic materials possess poor biocompatibility. Polydopamine (PDA) is the main component of the natural biological pigment melanin, and nanomaterial derived from PDA has excellent stability, biodegradability, biocompatibility and photothermal conversion characteristics [32].

**Fig. 2:**
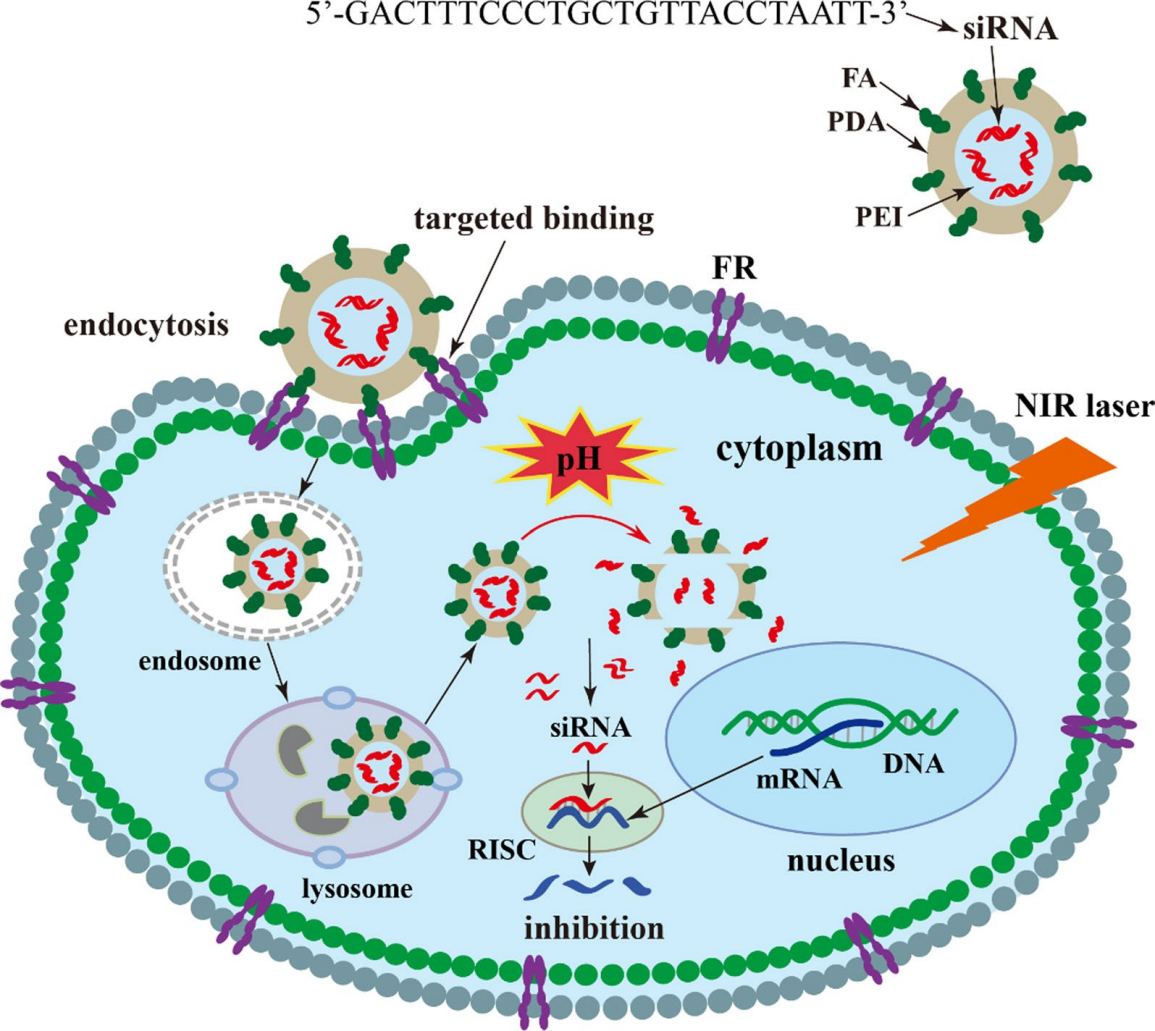
Schematic diagram of siRNA/PEI (polyethylenimine)-loaded and FA (folic acid)-modified PDA (polydopamine) nanomedicine used for knocking down the oncogene ROC1

In the present study, a siRNA/PEI-loaded FA-modified PDA nanomedicine was developed (Fig. 2). This genetic nanomedicine enters tumor cells via FA and its receptor mediated endocytosis. Subsequently, siRNA is released from the PDA nanomedicine into the tumor microenvironment in a pH-controlled manner. By knocking down the oncogene ROC1, suppressing the Neddylation modification process, inducing the accumulation of apoptotic and DNA damage factors, this genetic nanomedicine can inhibit the growth of liver cancer. Importantly, the PDA nanomedicine has superior therapeutic efficacy in the treatment of liver cancer when combined with NIR photothermal therapy. This biodegradable organic nanomedicine with targeted delivery shows good prospects for clinical application.

## Materials and methods

### Materials

Polyethylenimine (PEI, MW 10000), dopamine hydrochloride and folic acid (FA) were purchased from Macklin. N-(3-Dimethylaminopropyl)-Nʹ-ethylcarbodiimide hydrochloride (EDC), N-hydroxysuccinimide (NHS) and Hoechst 33258 were purchased from Sigma. The other chemical reagents used in this study were all analytically pure. Cy3-siRNA was purchased from Shanghai GenePharma Co., Ltd. LysoTracker® Green was purchased from Beyotime Biotechnology. BCA protein assay kits were purchased from Beyotime Biotechnology. Anti-ROC1, anti-Cullin1 (anti-Cul1), anti-Cullin5 (anti-Cul5), anti-ATF4, anti-P-H2AX, anti-cleaved caspase 3 and anti-Ki-67 antibodies were purchased from Abcam. CCK-8 (Cell Counting Kit-8) was purchased from MedChemExpress. An Annexin V FITC apototic kit was purchased from BD Biosciences.

### Synthesis of the siRNA/PEI-loaded FA-modified PDA nanomedicine

An alkaline alcohol aqueous solution was used to synthesize the PDA nanomedicine. Briefly, 625 μl of PEI (1 mg/ml) and 625 μl of Cy3-siRNA (20 μM) were incubated at room temperature for 30 min. Another mixture consisting of 3 ml of DEPC (diethylpyrocarbonate) water, 1.2 ml of ethanol, and 15 μl of NH_4_OH was stirred for 30 min. Then, the siRNA/PEI mixture was added to an aqueous alcohol solution including ammonia. Subsequently, 30 mg of dopamine hydrochloride was added to the mixture. The solution turned colorless and then dark during the reaction, indicating the continuous formation of PDA NPs. The reaction mixture was stirred overnight. Finally, the nanomedicine was collected by centrifugation at 10,000 rpm for 10 min to remove unloaded siRNA/PEI.

For the modification of the nanomedicine with the tumor-targeting FA moiety, the nanomedicine was first dispersed in 1 ml of phosphate-buffered saline (PBS) (10 mM, pH 7.0). Subsequently, 5 mg of FA was added to the FA/nanomedicine mixture. Next, 2 mg of EDC and 2 mg of NHS were added, and the mixture was stirred for 2 h. Finally, the FA-modified nanomedicine was collected by centrifugation at 10,000 rpm for 10 min, washed once with ultrapure water in a centrifuge, dispersed in 1 ml of DEPC water and stored at 4 °C until use. Similarly, blank PDA NPs were synthesized using the abovementioned method, except for the addition of Cy3-siRNA.

### Characterization of the PDA NPs

Transmission electron microscopy (TEM) and dynamic light scattering (DLS) were used to investigate the morphology and zeta potential of the prepared NPs. The samples were placed onto copper grills covered with nitrocellulose. The grid was allowed to dry at room temperature and then examined using TEM (JEM-1230, JEOL, Japan). As for the size distribution and zeta potential analysis, 1 mg/ml of samples were detected using a Malvern ZetaSizer Nano ZS90 particle size analyzer. For the infrared spectrum, 3 ml of sample was analyzed using a FTIR analyzer.

### Encapsulation efficiency of Cy3-siRNA in the PDA nanomedicine

On the basis of a standard curve (absorbance vs concentration) of Cy3-siRNA (553 nM), Cy3-siRNA-loaded PDA nanomedicine was dissolved in 0.1 M NaOH solution to measure the encapsulation efficiency of Cy3-siRNA. The encapsulation efficiency of Cy3-siRNA was 78.3 ± 4.6%, which was determined as the ratio of the encapsulation amount of Cy3-siRNA to the total amount of Cy3-siRNA added to the nanomedicine.

### Cell culture and cell viability

Human liver normal QSG-7701 cells and Huh7 and SK-Hep-1 liver cancer cells were purchased from the Cell Center of the Chinese Academy of Medical Sciences. All cells were cultured in DMEM (HyClone, Logan, UT) containing 10% fetal bovine serum (Biochrom AG, Berlin, Germany) and 1% penicillin–streptomycin solution at 37 °C with 5% carbon dioxide.

For the cell viability assays, cells were plated in 96-well plates at a density of 2 × 10^3^ cells per well in quadruplicate sets and cultured overnight. After incubation with different reagents for 48 h, the cells were subjected to CCK-8 assay according to the manufacturer’s specifications.

### Response of the Cy3-siRNA-loaded PDA nanomedicine to stimulus

For pH-responsive release, the Cy3-siRNA-loaded nanomedicine was explored on the basis of the optical signal emitted by Cy3-siRNA. First, 10 mg of Cy3-siRNA-loaded PDA nanomedicine was dissolved in 10 ml of 1× PBS buffer (pH = 7.4) and then divided into 10 aliquots with each containing 1 ml of nanomedicine solution. Five of the aliquots served as the control group, and the other five aliquots were treated with NIR irradiation (808 nm, 2 W∙cm^−2^, for 5 min). Simultaneously, 10 mg of Cy3-siRNA-loaded PDA nanomedicine was dissolved in 10 ml of 1 × PBS buffer (pH = 6.2) and divided into 10 aliquots containing with each containing 1 ml of the nanomedicine solution. Five of the aliquots were used as the pH (pH = 6.2) release group, and the other five aliquots were used as the pH (pH = 6.2) release with laser irradiation group. Then, four samples from each of these different groups were precipitated at predetermined time intervals, and Cy3-siRNA release in the supernatant was measured at 553 nm by ultraviolet spectrophotometry according to the standard curve.

### Targeted effect and lysosomal localization of the Cy3-siRNA-loaded FA-modified nanomedicine

For analysis of the targeting effect of FA and lysosomal localization of the genetic nanomedicine, QSG-7701, Huh7 and SK-Hep-1 cells were seeded in confocal dishes at 2 × 10^5^ cells per well and cultured for 24 h. For FA competition experiments, Huh7 and SK-Hep-1 cells were first incubated with 1 mM of FA for 1 h. Then, the cells were incubated with 0.5 mg/ml of Cy3-siRNA-loaded FA-modified PDA nanomedicine for 4 h. For lysosomal localization, the cells were first incubated with 0.5 mg/ml of Cy3-siRNA-loaded FA-modified PDA nanomedicine for 4 h. Then, the cells were incubated with 100 nM of lysosomal green fluorescent probe for 1 h. Subsequently, the samples were washed with PBS and fixed with 4% paraformaldehyde for 30 min. After the cells were washed with PBS, 0.5 μg/ml Hoechst 33258 was used to stain the cell nuclei for 5 min. Finally, after the dyed cells were washed with ultrapure water and dried, the cells was observed in the blue (Hoechst), red (Cy3-siRNA) and green (lysosomal fluorescent probe) channels of a fluorescence microscope (Leica).

### Knockdown efficiency and mechanism of action of the genetic nanomedicine

For the analysis of knockdown efficiency, 2 × 10^5^ Huh7 and SK-Hep-1 liver cancer cells were first seeded in 6-well plates and cultured for 24 h. Subsequently, the cells were incubated with Cy3-siRNA-loaded (2 μg/ml, 5ʹ-GACTTTCCCTGCTGTTACCTAAT-3ʹ) genetic nanomedicine with serum-free medium for 6 h, and the Huh7 and SK-Hep-1 liver cancer cells were further incubated in serum-containing medium for 72 h. Then, the cells were collected and washed three times with PBS and lysed with cell lysis buffer. The protein concentration of the lysates was determined sing a BCA protein assay kit. In total, 20 µg of protein from each sample was loaded into wells made in an SDS-PAGE gel, and electrophoresis was performed. To separate out ROC1, a 15% of SDS-PAGE gel was used. A 7.5% of SDS-PAGE gel was used to separate Cullin1 and Cullin5. In addition, 10% and 15% of SDS-PAGE gels were used for separating out ATF4 and P-H2AX, respectively. After separation, the proteins were transferred to nitrocellulose membranes, which was blocked in blocking buffer (containing 5% of nonfat dry milk) at room temperature for 1 h. Subsequently, the membrane was incubated overnight with primary antibodies at 4 °C, then with secondary antibody at room temperature for 2 h, and subsequently washed three times with TBST buffer. Finally, the bands in the membranes were visualized using ECL reagent and detected with a BIORAD ChemiDocTM Touch imaging system.

### In vitro antitumor efficacy of the genetic nanomedicine

For cell viability assays, 2 × 10^3^ Huh7 or SK-Hep-1 cells were seeded in 96-well plates at 2 × 10^3^ cells per well in quadruplicate and cultured overnight. After incubation with 200 μl (2 μg/ml) of siRNA- or siNT (nontargeted siRNA)-loaded nanomedicine for 48 h, the cells with siRNA- or siNT-loaded nanomedicine were treated by laser irradiation (808 nm, 2 W∙cm^−2^) for 5 min. Next, the cells were subjected to CCK-8 assay according to the manufacturer’s specifications.

For apototic analyses, 2 × 10^5^ Huh7 or SK-Hep-1 cells were first seeded in 6-well plates and cultured for 24 h. Then, the cells were incubated with 3 ml (2 μg/ml) of siRNA- or siNT-loaded nanomedicine for 48 h, and those with siRNA- or siNT-loaded nanomedicine were subjected to laser irradiation. Next, the cells were harvested and resuspended in Annexin V binding buffer and stained with Annexin V-FITC and PI at 37 °C for 5 min. Finally, the cells were analyzed with a flow cytometer.

### In vivo antitumor efficacy of siRNA-loaded PDA nanomedicine

For animal experiments, 6-week-old male nude mice (weight approximately 20 g) were purchased from Shanghai SLAC Laboratory Animal Co., Ltd. The nude mice were housed in a pathogen-free environment at the animal center at Shanghai University of Traditional Chinese Medicine according to the guidelines of the Institutional Animal Care and Use Committee (PZSHUTCM200710002).

The nude mice were first subcutaneously injected with 5 × 10^6^ live Huh7 cancer cells into the left shoulder. After initial tumor establishment (~ 50 mm^3^) for approximately 7 days, the tumor-bearing mice were randomly separated into 6 groups (n = 6). Then, the mice in different groups were treated with 100 μl of saline, NIR irradiation (808 nm, 2 W∙cm^−2^, 5 min), blank PDA NPs (~ 2 mg/ml), blank PDA NPs with NIR irradiation, siRNA-loaded nanomedicine (2 mg/kg) or the genetic nanomedicine with NIR irradiation every third day; injections were made through the tail vein. Before each treatment, tumor volume and body weight were recorded. Tumor volume was measured using a vernier caliper and calculated as follows: tumor size (mm^3^) = (length × width^2^)/2. After approximately three weeks of treatment, all mice in the six groups were sacrificed, and tumors were collected for tumor burden analysis.

### Immunohistochemical staining

Tumors and various organs obtained from each mouse group were fixed overnight with 5 ml of formalin, dehydrated in ethanol, embedded in paraffin, and sectioned (5 μm). Next, slides were deparaffinized in xylene and ethanol and rehydrated in water. For H&E staining, various organs were incubated with hematoxylin for 5 min and eosin for 1 min. As for the staining of antibodies, antigen retrieval was performed by heating the sections in a microwave for 30 min in sodium citrate buffer (pH = 6.0) for the tumors. The slides were then quenched in hydrogen peroxide (3%) to block endogenous peroxidase activity and washed with TBST buffer. Subsequently, the samples were incubated with primary antibodies overnight at 4 °C and then subjected to analysis with a SuperPicTure™ polymer detection kit (Life Technologies) according to the manufacturer’s instructions. Antibodies against ROC1, cleaved caspase 3 and Ki-67 were used for this analysis.

### Statistical analysis

Statistical analysis results are presented as the means ± standard deviation (SD). Furthermore, a statistically significant difference between two groups was analyzed by hypothesis testing with two-sample t tests, and significance is indicated by *p < 0.05, **p < 0.01 and ***p < 0.001. Moreover, p < 0.05 was considered statistically significant in all analyses (95% confidence level).

## Results and discussion

### Anti-liver cancer target ROC1 (RBX1)

To improve the therapeutic efficacy of the PDA nanomedicine, gene therapy combined with photothermal therapy was applied for the treatment of liver cancer. As shown in Fig. 2, a biodegradable PDA nanomedicine was prepared via the classical Stöber method (polymerization of dopamine hydrochloride in a basic alcohol-water solution) [33]. Subsequently, the FA molecule used for targeting cancer tissues was added the surface of the PDA nanomedicine through an EDC/NHS-mediated covalent coupling reaction involving the amino groups of PDA and the carboxyl groups of FA. Then, the genetic nanomedicine was delivered into tumor cells by FA via receptor-mediated endocytosis. Subsequently, Cy3-siRNA was released from the PDA nanomedicine in the acidic tumor microenvironment (the accumulation of acidic metabolites (lactates and protons) produced by the high-rate aerobic glycolysis of tumor cells). Finally, the genetic nanomedicine knock down the oncogene ROC1 (E3) and suppressed the Neddylation pathway, and its effect was also combined with that of photothermal therapy to efficiently inhibit the growth of liver cancer.

**Fig. 3:**
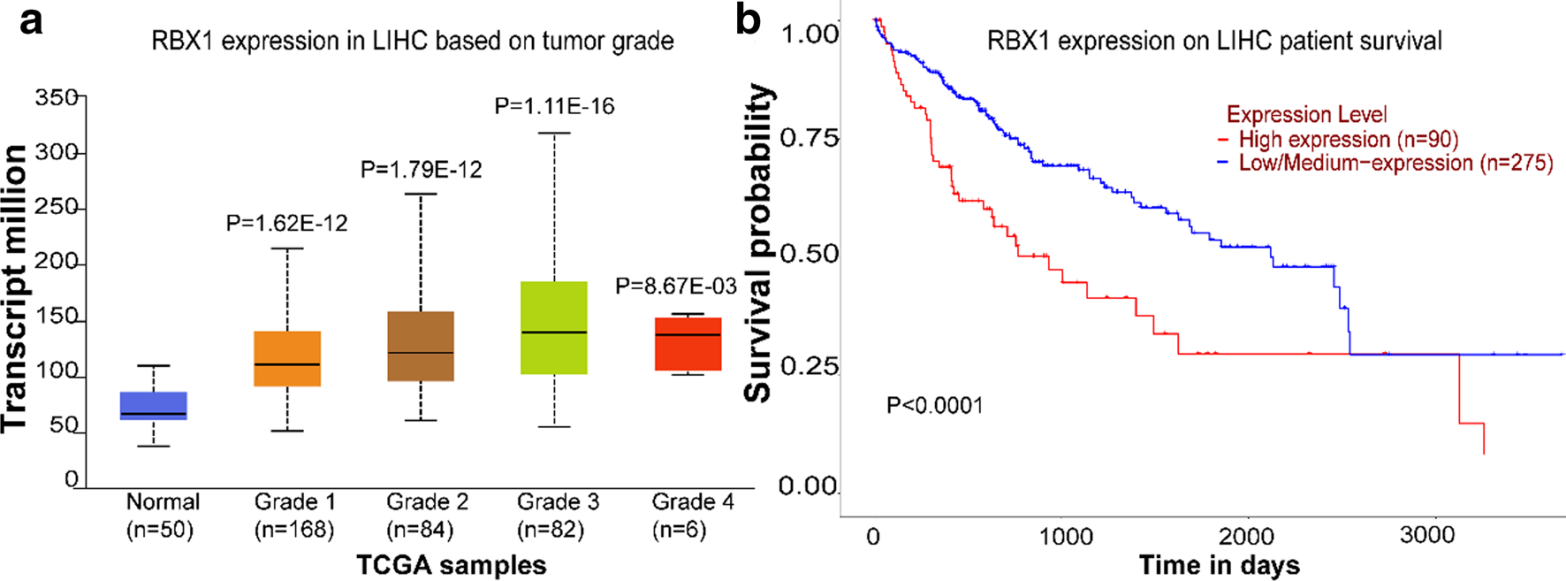
Overexpressed ROC1 correlated with tumor grade and predicted poor survival of liver cancer patients (TCGA analysis in UALCAN). **a** The expression of ROC1 in liver cancer patients was positively correlated with tumor grade. **b** Kaplan–Meier curves were analyzed to determine the overall survival rate of liver cancer patients according to the high expression and low expression of ROC1 (p < 0.0001)

To evaluate the role of ROC1 in the occurrence and development of liver cancer, the expression data of ROC1 in human liver cancer patients obtained from the UALCAN (TCGA) database were analyzed. As shown in Fig. 3A, the expression of ROC1 was found to be positively correlated with the degree of liver hepatocellular carcinoma malignancy. Moreover, as shown in Fig. 3B a Kaplan–Meier analysis revealed that liver cancer patients with high ROC1 expression showed worse overall survival than those with low ROC1 expression, indicating that higher expression of ROC1 can lead to a later tumor stage and a shorter survival rate of liver cancer patients. Altogether, overexpressed ROC1 serves as a serious risk factor for liver cancer patients, while knocking down ROC1 can inhibit the growth of liver cancer and prolong the survival time of patients with liver cancer.

### Synthesis and characterization of the PDA NPs

**Fig. 4:**
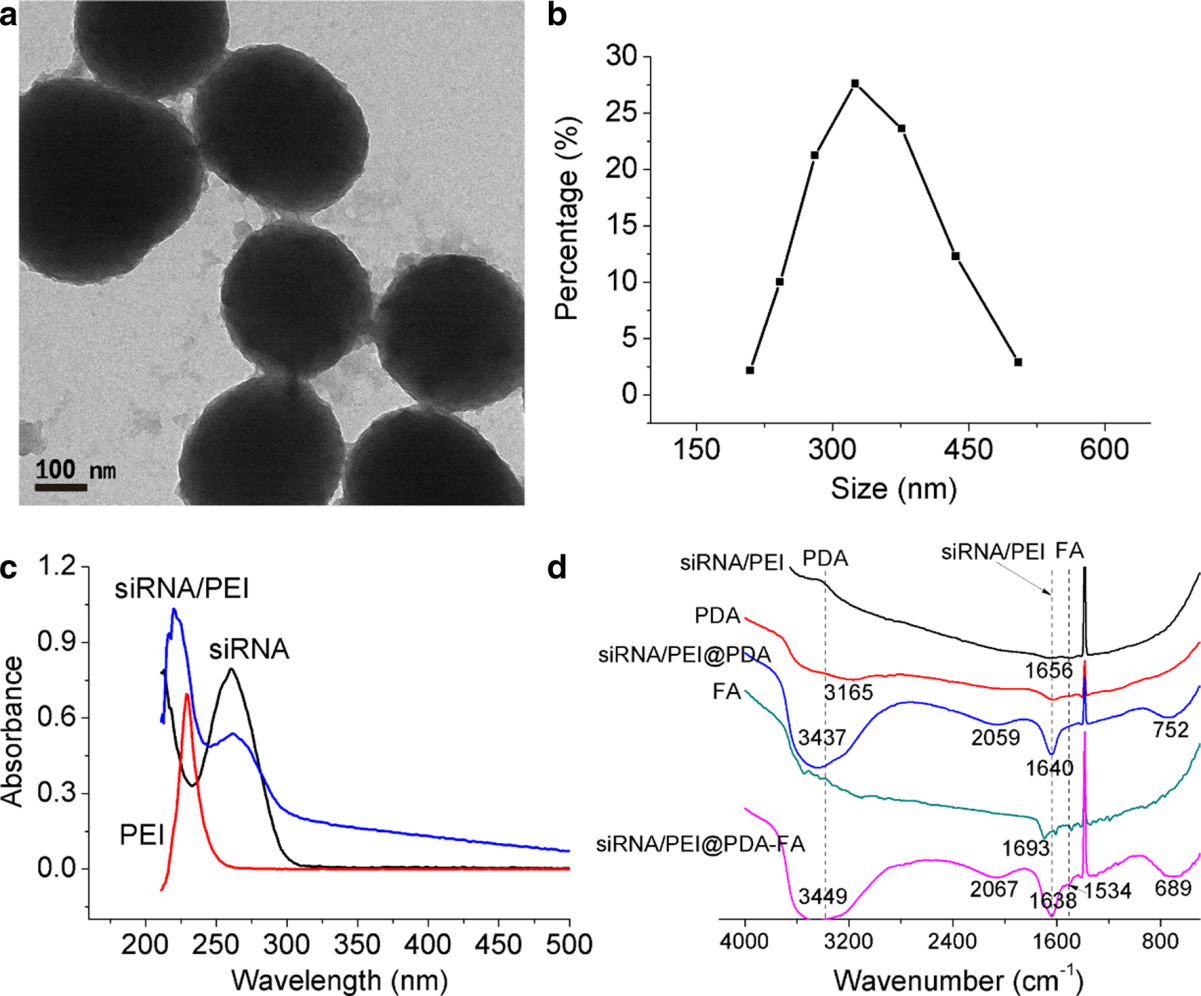
Characterizations of PDA NPs. **a** Transmission electron microscopy (TEM) image, scale bar: 100 nm. **b** Particle size distribution analysis performed with dynamic light scattering (DLS). **c** Ultraviolet absorption spectrum of siRNA/PEI. **d** Infrared (FTIR) spectrum of siRNA/PEI@PDA and FA modified siRNA/PEI@PDA

To construct an FA-modified and pH-responsive genetic nanomedicine in this study, PDA NPs were prepared through an easy three-step method. Cy3-siRNA and PEI were first formed into NPs through electrostatic binding. Subsequently, siRNA/PEI was encapsulated with dopamine hydrochloride in an alkaline alcohol aqueous solution. Then, the nanomedicine surface was modified by FA through a coupling reaction involving EDC/NHS. As shown in Fig. 4A, transmission electron microscopy (TEM) images revealed that the obtained PDA NPs have a spherical morphology. As shown in Fig. 4B, the size distribution curves suggest that most of the PDA NPs have a diameter of 320 nm. The stability of the blank PDA NPs was also monitored. As shown in Additional file 1: Figure S1, diameter changes in PDA NPs were negligible in PBS and 10% serum during 15 days, indicating the PDA NPs possesses excellent stability.

To verify the synthesis of the PDA nanomedicine and the modification of target molecule, ultraviolet absorption and infrared (FTIR) experiments were performed. As shown in Fig. 4C, the ultraviolet absorption of siRNA/PEI had characteristic peaks of 260 nm (siRNA) and 225 nm (PEI), indicating siRNA/PEI NPs were successfully synthesized. As shown in Fig. 4D, the infrared spectrum of siRNA/PEI loaded PDA possessed characteristic peaks of 1640 cm^−1^ (siRNA/PEI, NH bending vibration) and 3437 cm^−1^ (PDA, stretching vibration of phenolic hydroxyl and NH groups), suggesting siRNA/PEI was loaded into PDA nanomedicine [34]. In addition, 1694 cm^−1^ peak (C=O stretching vibration) of FA was free carbonyl group, but this peak shifted to 1638 cm^−1^ (C=O stretching vibration of amide peak (I)) of FA-modified siRNA/PEI@PDA. And FA-modified siRNA/PEI@PDA also had characteristic amide NH peak (II) at 1534 cm^−1^, indicating FA was successfully modified on the surface of PDA nanomedicine [35, 36]. The particle size and zeta potential of the siRNA/PEI NPs, siRNA/PEI-loaded PDA nanomedicine and FA-modified siRNA/PEI-loaded PDA nanomedicine were also measured. As shown in Additional file 1: Figure S2A, the particle size of the siRNA/PEI NPs was 145 nm. The particle sizes of the siRNA/PEI-loaded PDA nanomedicine and FA-modified siRNA/PEI-loaded PDA nanomedicine were 310 nm and 320 nm, respectively. As shown in Additional file 1: Figure S2B, the zeta potentials of the siRNA/PEI NPs, siRNA/PEI-loaded PDA nanomedicine and FA-modified siRNA/PEI-loaded PDA nanomedicine were 25 mV, 34 mV and 26 mV, respectively. These results indicate that siRNA/PEI was successfully embedded in the PDA nanomedicine and that the targeted molecule FA was effectively added on the surface of the PDA nanomedicine.

### Controlled release triggered by pH and the photothermal conversion ability of the PDA nanomedicine

**Fig. 5:**
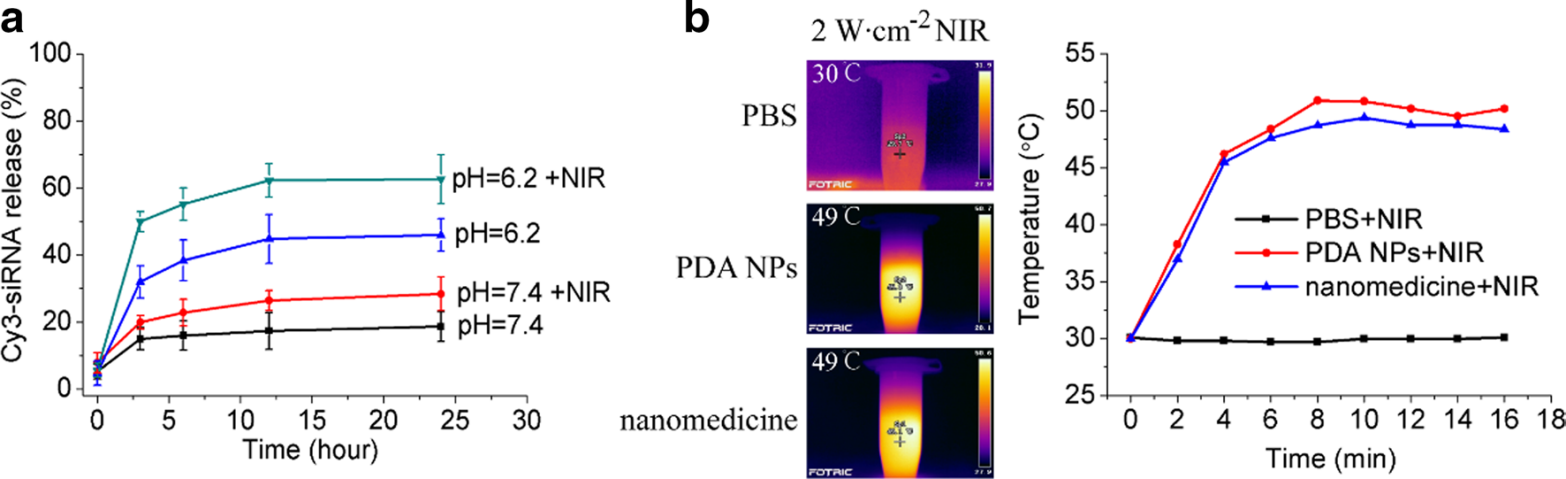
pH-controlled release and optothermal response of Cy3-siRNA-loaded PDA nanomedicine. **a** Cy3-siRNA release from Cy3-siRNA-loaded PDA nanomedicine (0.5 mg/ml) after treatment with PBS buffer at pH = 6.2 in the absence and presence of 808 laser irradiation (2 W∙cm^−2^, 5 min); error bars represent the mean ± SD (standard deviation, n = 3). **b** Pictures and temperature curves of the PBS buffer, blank PDA NPs, and Cy3-siRNA-loaded PDA nanomedicine after treatment with 808 nm laser irradiation

The pH level of tumor tissues (6.5–6.9) is generally lower than that of paracarcinoma tissues (7.2–7.4), which is caused by upregulated glycolysis, which produces lactates and protons in tumor environments. The pH-responsive release of the siRNA-loaded PDA nanomedicine was studied under low pH conditions (pH = 6.2) without or with NIR laser irradiation. As shown in Fig. 5A, the release rate of Cy3-siRNA was only 17% in the control group (PBS buffer with pH = 7.4). This minor leakage indicated that the nanomedicine was relatively stable under normal conditions. After 808 nm laser treatment (2 W∙cm^−2^, 5 min), the release of Cy3-siRNA gradually increased to 26%, which may have been due to the thermal expansion efficacy caused by NIR laser irradiation. In addition, the release of Cy3-siRNA increased tremendously, to 46%, at pH = 6.2. This pH-dependent release may have been due to the pH sensitivity of the PDA nanocarrier [37]. Importantly, the release of Cy3-siRNA maximally increased to 63% after treatment with the 808 nm laser at pH = 6.2, indicating that Cy3-siRNA release was further enhance under diverse pH conditions and NIR light. Importantly, most Cy3-siRNA was released from the PDA nanomedicine during the first 6 h, revealing that the anticancer agent can be easily released from the nanomedicine in an acidic tumor microenvironment in the presence of an NIR stimulus.

For photothermal therapy, the photothermal response of the PDA nanomedicine was also explored. As shown in Fig. 5B, the 808 nm laser had little influence on the PBS buffer. The temperatures of the blank PDA nanocarrier and Cy3-siRNA-loaded PDA nanomedicine increased similarly over time. After approximately 10 min of irradiation, the temperatures reached a plateau (approximately 48 °C). In addition, the photostability of the PDA NPs was measured. As shown in Additional file 1: Figure S3, the photothermal conversion efficiency of the PDA nanomedication was basically unaffected after five rounds of ON/OFF irradiation cycles, indicating that the PDA NPs have excellent photothermal stability. Collectively, the results indicate that siRNA-loaded PDA NPs can be used in photothermal therapy for the treatment of liver cancer.

### Biocompatibility of the blank PDA nanocarrier

Dopamine (a natural melanin) has been shown to have good biocompatibility as a nanocarrier [38]. In theory, the metabolic products of dopamine are biodegradable homovanillic acid and trihydroxyphenylacetic acid [39]. As shown in Additional file 1: Figure S4, the blank PDA NPs induced no obvious cytotoxicity in QSG-7701 normal liver cells or Huh7 and SK-Hep-1 liver cancer cells, indicating that the PDA NPs have excellent biocompatibility.

In the present study, PDA NPs were used not only as nanocarriers but also as photothermal therapy agents. Hence, the influence of the 808 nm laser on cell growth was explored. As shown in the last panel of Additional file 1: Figure S4, the 808 nm laser emitting at 2 W∙cm^−2^ for 5 min had a negligible influence on the proliferation of QSG-7701, Huh7 and SK-Hep-1 cells, revealing that the 808 nm laser had little influence on cell growth without photothermal agents.

### Targeted delivery and lysosomal localization of the PDA genetic nanomedicine

**Fig. 6:**
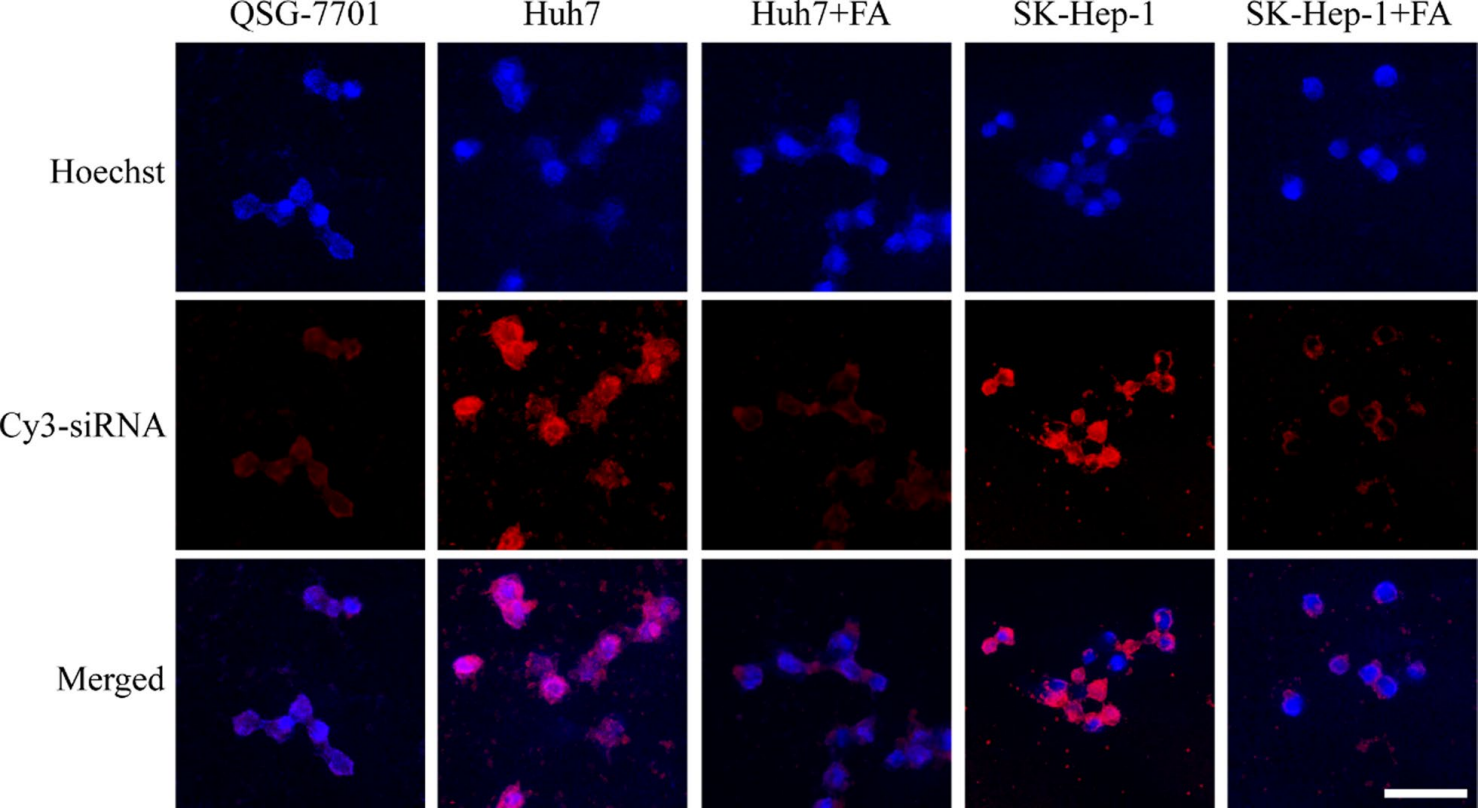
Fluorescence microscopy images of targeted delivery. QSG-7701 normal liver cells, Huh7 and SK-Hep-1 liver cancer cells incubated with FA-modified Cy3-siRNA-loaded PDA nanomedicine for 4 h after incubation without or with 1 mM of FA for 1 h. The red color is Cy3-siRNA, the blue color is Hoechst, and the scale bar is 20 μm

To improve the therapeutic efficacy and reduce the off-target effect of the genetic nanomedicine, FA was used as a targeting molecule added to the surface of the PDA nanomedicine. To verify the targeting effect of FA, QSG-7701 normal liver cells and Huh7 and SK-HEP-1 hepatocellular carcinoma cells were incubated with Cy3-siRNA-loaded FA-modified PDA nanomaterials due to the optical signal of Cy3-siRNA. For FA competition experiments, Huh7 and SK-HEP-1 cells were first incubated with FA, then incubated with FA-modified genetic nanomaterials. As depicted in Fig. 6, fluorescence microscopy observation revealed a very little red fluorescence around QSG-7701 normal liver cells. This phenomenon was attributed to the nonspecific endocytosis. In comparison, the targeted genetic nanomedicine around the Huh7 and SK-Hep-1 liver cancer cells showed high levels of endocytosis, suggesting that the targeting of the nanomedicine to liver cancer cells was better than that to normal liver cells. However, there was less endocytosis of genetic nanomedicine in Huh7 and SK-Hep-1 cells after competition with FA. Taken together, these findings indicate that modification with FA as a targeting molecule can enable specific delivery anticancer drug siRNA to liver tumor cells. In addition, the cell internalization of this genetic NPs in Huh7 and SK-Hep-1 liver cancer cells were relatively high (over 90%) according to the results of fluorescence microscopy, indicating that most of the anticancer siRNA gene was transported into hepatocellular carcinoma cells.

The genetic nanomedicine enter cells by an endocytosis pathway, while its lysosomal escape is crucial for the subsequent oncogenic knockdown. Therefore, fluorescence microscopy images of lysosomal co-localization experiment of the genetic nanomedicine was performed. As shown in Additional file 1: Figure S5, most of the genetic nanomedicine was co-localized with lysosomes in the first 4 h, indicating the genetic nanomedicine still resided in the lysosome. While the co-localizations of the genetic nanomedicine and the lysosome were decreased after 12 and 24 h of incubation, demonstrating the genetic nanomedicine had escaped from the lysosome. The genetic nanomedicine entered liver cancer cells through FA and its receptor mediated endocytosis. Then, the nanomedicine reached in lysosome. Some of the nanomedicine might escape from lysosomes through the proton sponge effect due to its positive zeta potential (Additional file 1: Figure S2B). In addition, lysosome membrane becomes unstable upon internalization of nanomedicine, resulting in nanomedicine escape/relocalisation into the cytosol [40]. Because of the acidic microenvironment of lysosomes, siRNA/PEI might also be released from the PDA nanomedcine in lysosomes. Then, siRNA/PEI escaped from lysosomes through the proton sponge effect and lysosome membrane destabilization according to the report [21, 40].

### Knockdown efficiency of ROC1 using the designed genetic nanomedicine

**Fig. 7:**
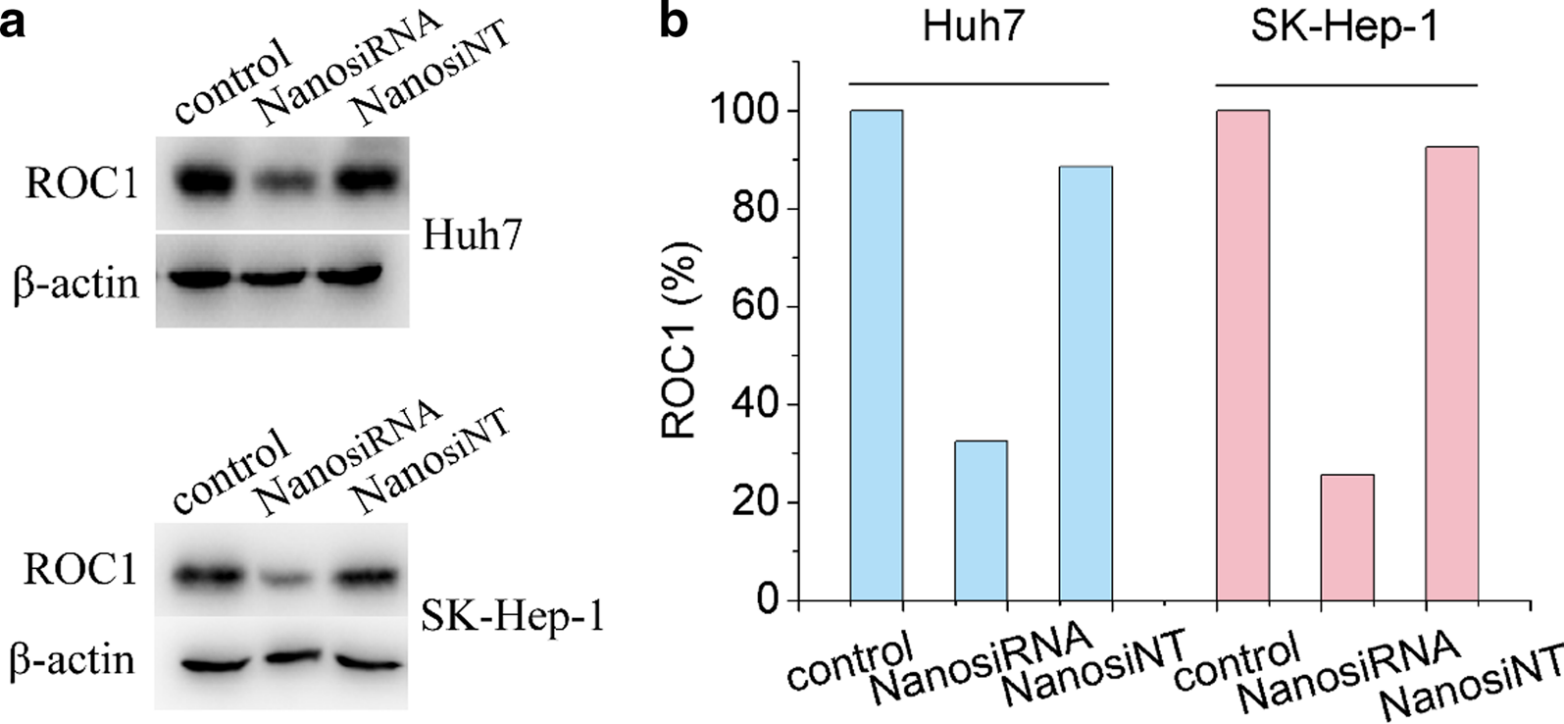
Efficiency of oncogene ROC1 knockdown in Huh7 and SK-Hep-1 liver cancer cells in vitro. **a** Western blot analysis of the knockdown efficiency of ROC1 in Huh7 and SK-Hep-1 liver cancer cells after incubation with FA-modified Cy3-siRNA-loaded PDA nanomedicine (containing 2 μg/ml siRNA) for 72 h, and for the genetic nanomedicine, β-actin was as an internal standard. **b** Grayscale analysis of the western blot bands using ImageJ software

The knockdown efficiencies of the designed genetic nanomedicine on the oncogene ROC1 in Huh7 and SK-Hep-1 liver cancer cells were detected by western blotting. As shown in Fig. 7, the ROC1 expression level in Huh7 cells with siRNA- and siNT (untargeted siRNA)-loaded nanomedicine was 33% and 89%, respectively. In addition, the ROC1 expression level in SK-Hep-1 cells with siRNA- and siNT-loaded nanomedicine was 27% and 91%, respectively. These results have demonstrated that genetic nanomedicine can effectively knock down the ROC1 level in both Huh7 and SK-Hep-1 liver cancer cells. In contrast, the random siRNA NT sequence did not have this effect. In summary, the genetic nanomedicine can efficiently deliver siRNA into liver cancer cells and silence the ROC1 oncogene.

### In vitro antitumor activity of the genetic nanomedicine

**Fig. 8:**
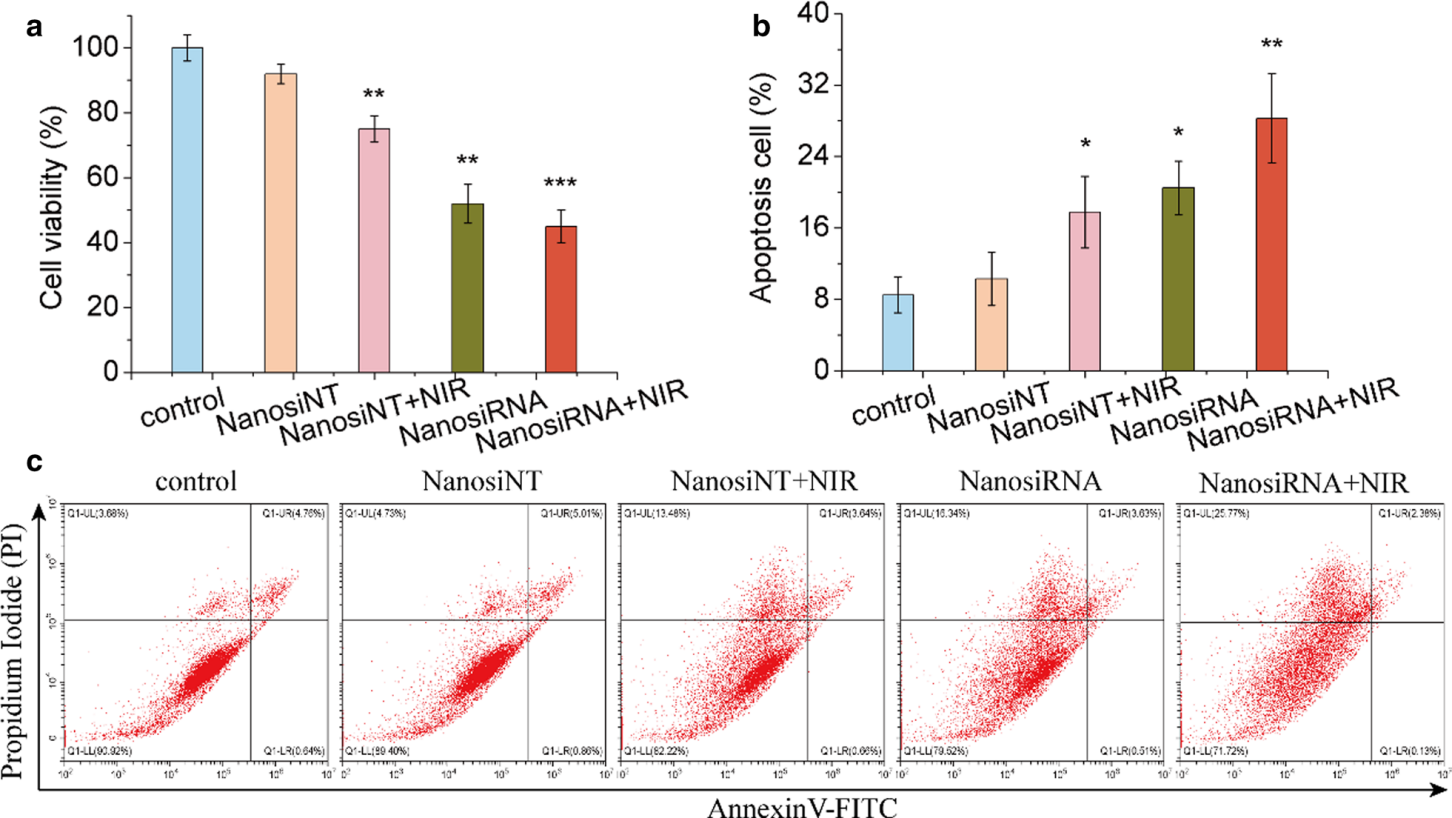
In vitro anticancer activity of the genetic nanomedicine combined with photothermal therapy in Huh7 liver cancer cells. **a** Viability of the Huh7 cells after incubation with siNT or siRNA-loaded PDA nanomedicine (containing 2 μg/ml siRNA or siNT) in the absence or presence of laser irradiation for 48 h. **b** Flow cytometric analysis of Huh7 cell apoptosis on the basis of Annexin V and FITC-PI staining. **c** Statistical analysis of the apoptotic cells corresponding to **b**. Error bars represent the mean ± SD (standard deviation, n = 3), *p < 0.05, **p < 0.01, ***p < 0.001

To explore the anticancer activity of the genetic nanomedicine, the proliferation and apototic rates induced by delivery of the genetic nanomedicine were analyzed in liver cancer cells. As shown in Fig. 8A, a random NT sequence had a negligible influence on the proliferation of Huh7 cells (7%), while siNT nanomedicine plus irradiation with an 808 laser effectively inhibited the viability of Huh7 cells (25%). Interestingly, knocking down ROC1 without or with laser irradiation could further inhibit the proliferation of Huh7 cell (48% and 55%). In addition, an apototic analysis was also performed. As shown in Fig. 8B, C, siNT nanomedicine plus laser irradiation induced the apoptosis of more Huh7 cells (18%) than did the siNT nanomedicine (9%). The genetic nanomedicine leaded to more apoptosis of Huh7 cell (21%). While the genetic nanomedicine with 808 laser irradiation induced the greatest percentage of Huh7 cell to undergo apoptosis (28%). These results indicated that the genetic nanomedicine combined with NIR irradiation can inhibit the proliferation of Huh7 cells and promote their apoptosis by knocking down the oncogene ROC1.

The inhibitory activity of the genetic nanomedicine was also investigated in SK-Hep-1 liver cancer cells. As described in Additional file 1: Figure S6A–C, the random NT sequence had little influence on the proliferation or apoptosis of SK-Hep-1 cells. While siNT nanomedicine with laser irradiation inhibited the proliferation (28%) of SK-HEP-1 cells and promoted their apoptosis (16%). Notably, siRNA nanomedicine showed superior performance in inhibiting SK-HEP-1 cell proliferation (43%) and promoting their apoptosis (20%). Importantly, when combined with NIR irradiation, the inhibition ability (64%) and apototic efficacy (41%) of the genetic nanomedicine reached a maximum. Photothermal therapy mainly causes thermal ablation of cells. These results indicate that the FA-modified pH-responsive genetic nanomedicine shows excellent inhibitory efficacy on hepatocellular carcinoma cells in vitro when combined with NIR photothermal therapy.

### Inhibition of the Neddylation pathway by knocking down ROC1

**Fig. 9:**
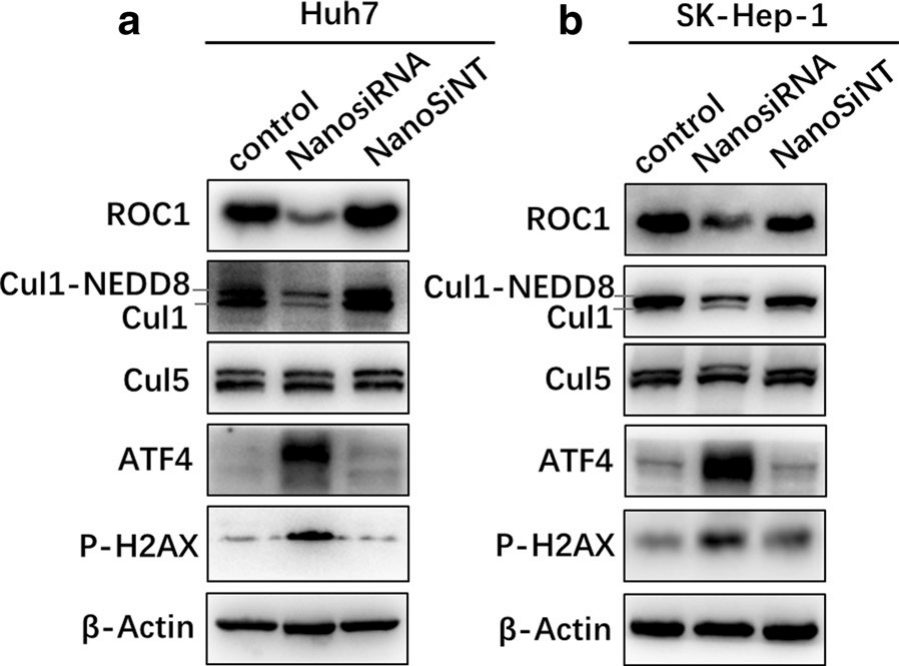
ROC1 knockdown induced downregulation of the Neddylation levels of Cullin1 and accumulation of apoptosis factor (ATF4) and DNA damage (P-H2AX) in **a** Huh7 liver cancer cells and **b** SK-Hep-1 liver cancer cells

To investigate the efficiency of ROC1 (E3) knockdown for the inhibition of liver cancer cell proliferation, the mechanism was studied by western blot analysis. As shown in Fig. 9A, the genetic nanomedicine significantly inhibited the Neddylation modification of Cul1 but had little influence on the Neddylation modification of Cul5 in Huh7 cells. This outcome was a result of the role of Cul1 as a ROC1 substrate (RBX1, E3), while Cul5 is a substrate of ROC2 (RBX2/SAG, E3) [41]. Moreover, ROC1 knockdown lead to upregulated ATF4 expression, which can promote the apoptosis of tumor cells [42]. In addition, the accumulation of DNA damage factor (P-H2AX) was also observed. However, there was no obvious difference in the NanosiNT group, indicating that the random NT sequence did not have the same impact as the nanomedicine. Furthermore, similar results were also obtained in SK-Hep-1 liver cancer cells, as shown in Fig. 9B. Taken together, these results indicate that ROC1 knockdown induces the accumulation of the apoptosis factor ATF4 and DNA damage factor P-H2AX by downregulating the Neddylation pathway. Therefore, knockdown of ROC1 inhibits the growth of liver cancer cells mainly through apoptosis pathway, as well as the impact of DNA damage.

### In vivo antitumor activity of the genetic nanomedicine

In vivo biodistributions of free Cy3-siRNA, Cy3-siRNA-loaded PDA nanomedicine without and with FA modification were monitored using an in vivo imaging system. As shown in Additional file 1: Figure S7A, the genetic nanomedicine possessed higher fluorescent signal than that of free Cy3-siRNA in tumor at 24 h. While the genetic nanomedicine with FA modification has stronger fluorescent signal in tumor and lower fluorescent signal in other organs than that of the genetic nanomedicine without FA modification at 24 h. As shown in Additional file 1: Figure S7B–D, the highest fluorescent signal of free Cy3-siRNA was observed in tumor at 12 h. While the highest fluorescent signals of the genetic nanomedicine without and with FA modification were detected in tumors at 24 h, indicating the genetic nanomedicine had the character of long-term circulation. Importantly, the genetic nanomedicine with FA modification emitted the lower fluorescence in liver and the higher fluorescence in tumor than that of the nanomedicine without FA modification, demonstrating it possesses good tumor targeting ability. These results reveal that the PDA nanomedicine with FA modification for targeting tumors can improve the therapeutic efficacy and reduce the potential side effects of liver cancer treatment.

The toxic and side effects of the NIR laser, blank PDA NPs, PDA NPs irradiated with an NIR laser, and genetic nanomedicine without and with photothermal treatment were studied in Huh7 tumor-bearing mice. As shown in Additional file 1: Figure S8, neither mouse death nor a significant drop in body weight was observed in any group during the monitoring period. Furthermore, H&E staining of major organs was also used to evaluate the side effects. Additional file 1: Figure S9 exhibited the heart, liver, spleen, lung, and kidneys of various groups remained the normal physiological morphologies, and no pathological changes were observed. Taken together, the genetic nanomedicine combining photothermal therapy didn’t produce serious toxicity or side effects in the tumor-bearing mice.

**Fig. 10:**
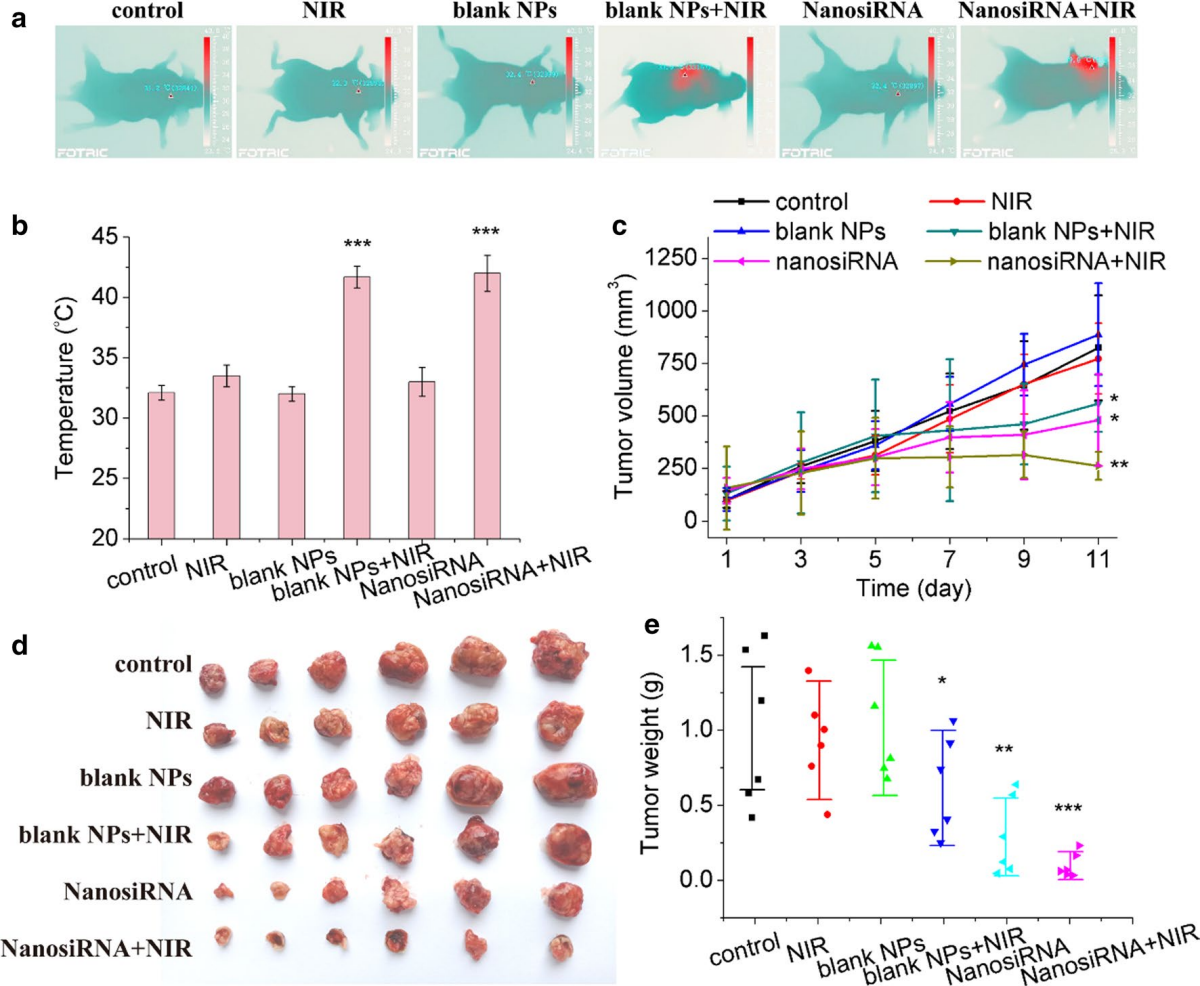
In vivo antitumor efficacy of the genetic nanomedicine combined with photothermal therapy. **a** Optothermal photos, **b** optothermal temperature changes, **c** relative tumor volume growth curves, **d** tumor photos and **e** tumor weights of the Huh7 tumors after systemic administration of saline only, NIR laser irradiation, blank PDA NPs, blank NPs with laser irradiation, siRNA-loaded nanomedicine and siRNA-loaded nanomedicine with laser irradiation. Error bars represent the mean ± SD (standard deviation, n = 6), *p < 0.05, ** < 0.01, ***p < 0.001

Subsequently, the antitumor activity of the genetic nanomedicine with photothermal therapy was explored. First, the photothermal effect of the NPs was recorded. As shown in Fig. 10A, B, for the groups treated with saline, blank PDA NPs and siRNA-loaded nanomedicine, the temperatures of the tumor region were 32.7, 31.9 and 32.8 °C, respectively. The temperatures increased to 33.4, 41.6, and 42.1 °C for the groups treated with NIR irradiation, blank NPs plus NIR irradiation (2 W∙cm^−2^, 5 min), and siRNA-loaded nanomedicine plus NIR irradiation, respectively. These results revealed that the PDA NPs exposed to the NIR laser increased the temperature of tumor tissue. More importantly, thermal ablation could induce subsequent photothermal therapy. Futhermore, the antitumor efficacies of the aforementioned groups were explored. As shown in Fig. 10C, the NIR laser group and blank PDA NP group showed an impact similar to that of the control group, indicating that laser irradiation had no significant influence on the tumor-bearing mice without photothermal reagents and that the PDA nanocarrier showed good biocompatibility. However, an NIR laser exposure of mice injected with blank NPs had a significant tumor inhibition effect, revealing that photothermal therapy can inhibit the growth of liver cancer. In addition, the genetic nanomedicine showed better antitumor activity, indicating that gene therapy can inhibit the growth of tumors by knocking down the ROC1 oncogene. Notably, the best performance was achieved by the mouse group that received the genetic nanomedicine and NIR radiation exposure, as indicated by the tumor volume in these mice shrinking remarkably over time. Tumor images (Fig. 10D) and tumor weight (Fig. 10E) were consistent with the tumor volume data. All these results demonstrate that the positive targeting to tumor tissues, pH-triggered and photothermally fast release of siRNA in the tumors, and the local hyperthermic ablation efficacy of PDA contributed to the excellent antitumor activity of the genetic nanomedicine.

**Fig. 11:**
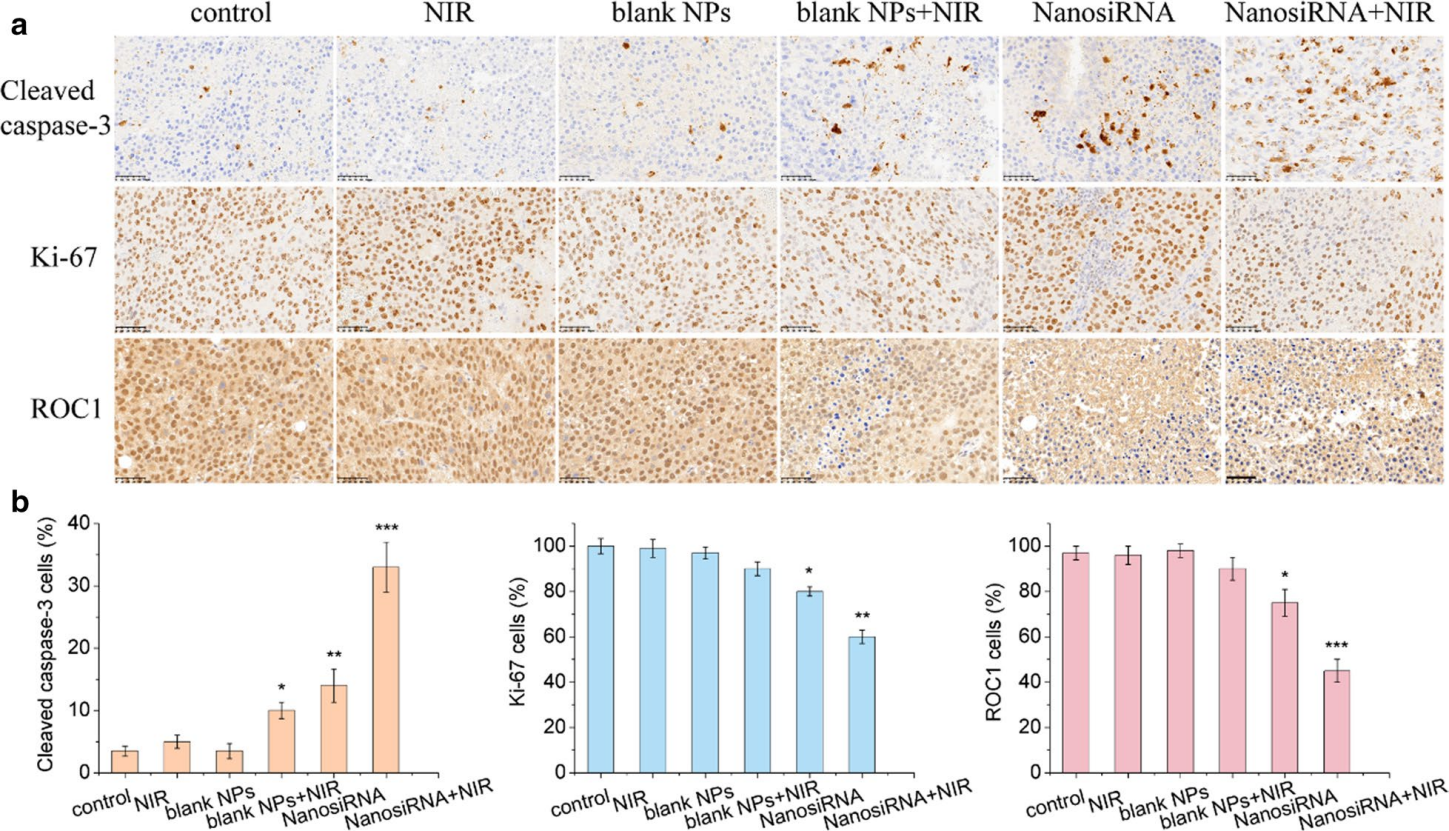
Immunohistochemical staining. **a** Immunohistochemical staining of cleaved caspase-3, Ki-67 and ROC1 in Huh7 tumors after systemic administration of saline only, NIR laser irradiation, blank PDA NPs, blank NPs with laser irradiation, siRNA-loaded nanomedicine and siRNA-loaded nanomedicine with laser irradiation. **b** Statistical results corresponding to **a**, error bars represent the mean ± SD (standard deviation, n = 6), *p<0.05, **<0.01, ***p<0.001.

Immunohistochemical results were also obtained to determine the anticancer efficacy of the genetic nanomedicine. As shown in Fig. 11, compared with the control group, the NIR laser and blank NP groups showed similar expression of the tumor proliferation factor (Ki-67) and apoptosis factor (cleaved caspase-3), indicating the excellent biocompatibility of the NIR light and blank PDA nanocarrier. The blank NPs with NIR laser exhibited more positive staining of cleaved caspase-3 and less positive staining of Ki-67, suggesting that the nanocarrier has photothermal conversion capability. In addition, the genetic nanomedicine treatment led to higher cleaved caspase-3 and lower Ki-67 expression levels, indicating that the genetic nanomedicine can inhibit tumor growth. The genetic nanomedicine plus NIR group exhibited the highest cleaved caspase-3 and the lowest Ki-67 levels, indicating its superior therapeutic efficacy. Notably, the siRNA nanomedicine administered without and with NIR laser irradiation reduced the expression of ROC1 in Huh7 tumors, demonstrating that the genetic nanomedicine can be used to treat liver cancer by knocking down the ROC1 oncogene and suppressing the Neddylation pathway. Considering the selective cytotoxicity of the genetic nanomedicine to FR and low pH-rich liver cancer cells, compared to normal cells, these results can likely be ascribed to the targeted delivery and the on-demand drug release triggered from the siRNA nanomedicine, as well as the combinatory effect of the genetic nanomedicine and photothermal therapy.

## Conclusions

In the TCGA databases, the high expression of ROC1 is highly correlated with the poor prognosis of liver cancer patients. In the present study, siRNA-loaded PDA nanomedicine with targeted delivery and a smart response as well as photothermal therapy capability was designed for knocking down the ROC1 oncogene. The genetic nanomedicine can be delivered to liver cancer cells through FA and is internalized through receptor-mediated cellular endocytosis. Low pH levels and NIR irradiation stimulate the on-demand release of the loaded anticancer agent, siRNA, at the tumor site. In vitro and in vivo experiments indicated that the genetic nanomedicine combined with photothermal therapy can powerfully inhibit the proliferation of liver cancer cells and promote their apoptosis. Moreover, the genetic nanomedicine knocks down ROC1, suppresses the Neddylation pathway, induces the accumulation of the apoptosis factor ATF4 and DNA damage factor P-H2AX. More importantly, this genetic nanoplatform provides a promising strategy for combining the nanomedicine with other genetic agents or anticancer drugs. Therefore, the present study illustrates the great potential of biodegradable PDA nanomedicines used in conjunction with gene treatment and/or photothermal therapy as effective therapies against various types of malignant tumors.

## Supplementary Information

**Additional file 1.** **Figure S1.** stability of nanomaterial, **Figure S2.** particle size and zeta potential, **Figure S3.** photostability, **Figure S4.** biocompatibility, **Figure S5.** lysosomes escape, **Figure S6.** *in vitro* anticancer activity in SK-Hep-1 cells, **Figure S7.** *in vivo* image of mice, **Figure S8.** mouse weight, Figure S9 H&E staining images.

## Data Availability

All data generated or analyzed during this study are included in this published article [and its additional files].
