# Supplementary material for: A polydopamine nanomedicine used in photothermal therapy for liver cancer knocks down the anti-cancer target NEDD8-E3 ligase ROC1 (RBX1)
Source: J Nanobiotechnology. 2021 Oct 15;19:323. doi: 10.1186/s12951-021-01063-4 (PMC8518243; doi:10.1186/s12951-021-01063-4)
Supplement: Supplementary file 1 — Additional file 1. Figure S1. stability of nanomaterial, Figure S2. particle size and zeta potential, Figure S3. photostability, Figure S4. biocompatibility, Figure S5. lysosomes escape, Figure S6. in vitro anticancer activity in SK-Hep-1 cells, Figure S7. in vivo image of mice, Figure S8. mouse weight, Figure S9 H&E staining images. [file 12951_2021_1063_MOESM1_ESM.doc]

**Supporting information**

**A polydopamine nanomedicine used in photothermal therapy for liver cancer knocks down the anti-cancer target NEDD8-E3 ligase ROC1 (RBX1)**

Zhanxia Zhang^1#,*^, Junqian Zhang^1#^, Jianhui Tian^1^, Hegen Li^2^

^1^Cancer Institute, Longhua Hospital, Shanghai University of Traditional Chinese Medicine, 725 Wanping South Road, Shanghai, 200032, China.

^2^Department of Medical Oncology, Longhua Hospital, Shanghai University of Traditional Chinese Medicine, 725 Wanping South Road, Shanghai, 200032, China.

^#^ These authors contributed equally to this work.

^*^Corresponding author: Zhanxia Zhang, zhanxiazhang@shutcm.edu.cn

**Keywords:** ROC1; Neddylation; siRNA-loaded nanomedicine; photothermal therapy; targeted delivery


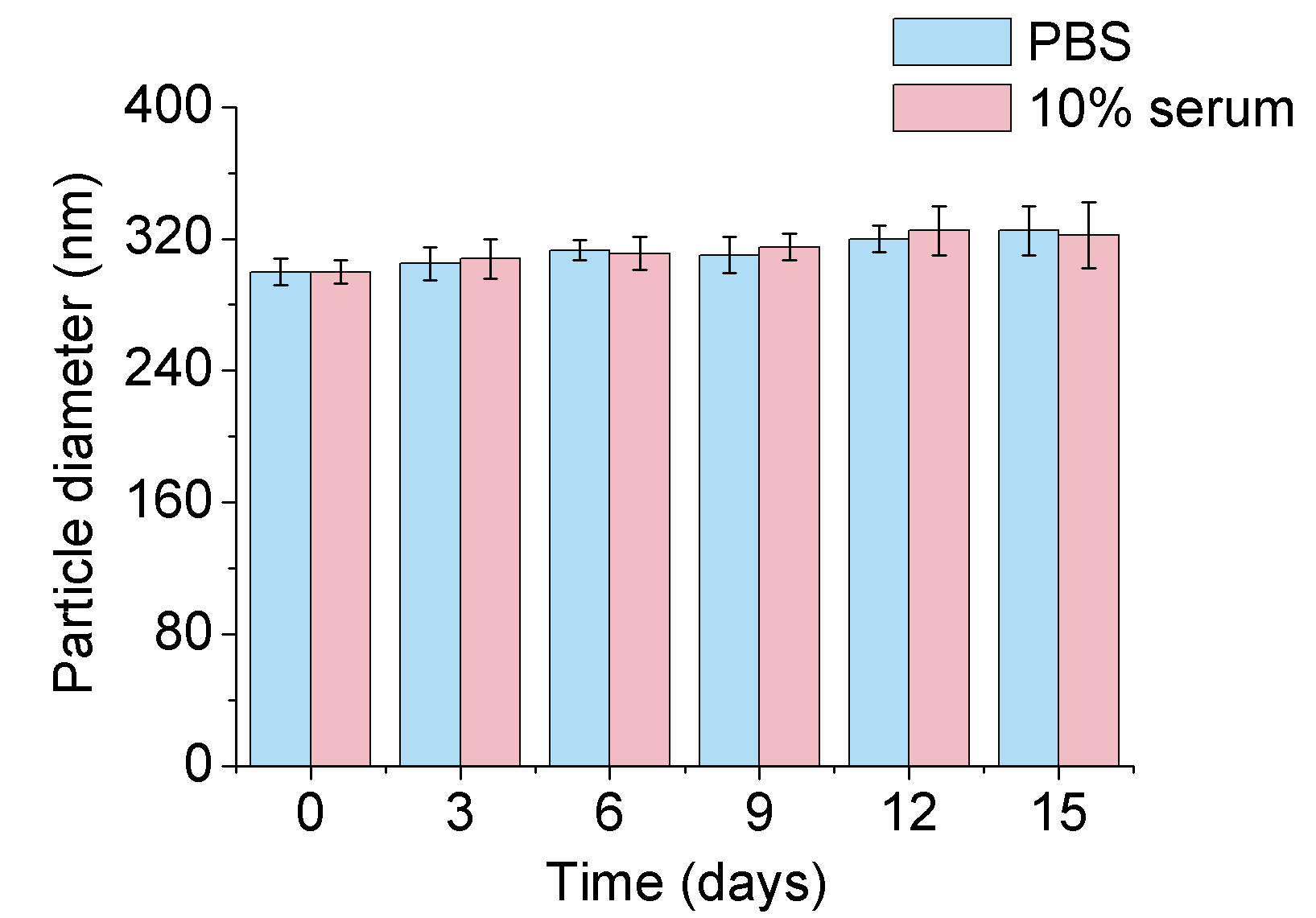


**Figure S1**. Stability of the PDA nanoparticles. Error bars represent mean ± SD (standard deviation, n = 3).

**
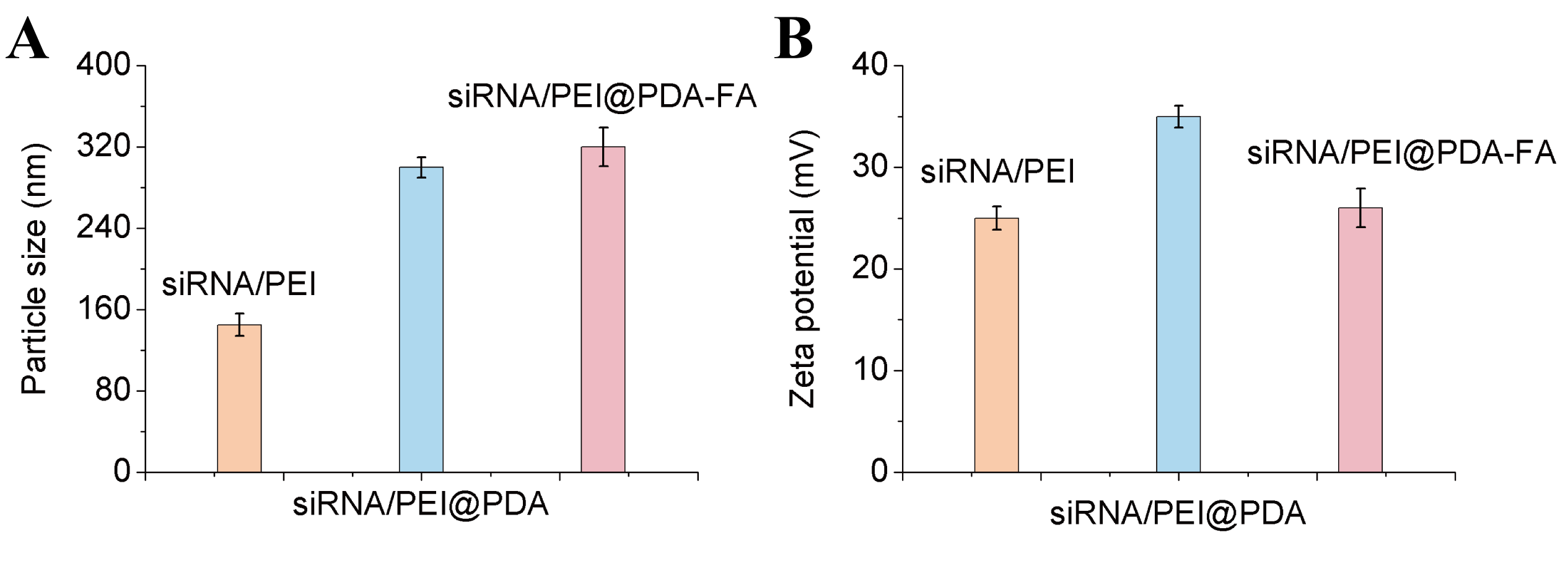
**

**Figure S2**. (A) Particle size and (B) zeta potentials of siRNA/PEI, siRNA/PEI@PDA and FA-modified siRNA/PEI@PDA nanomedicine. Error bars represent the mean ± SD (standard deviation, n = 3).

**
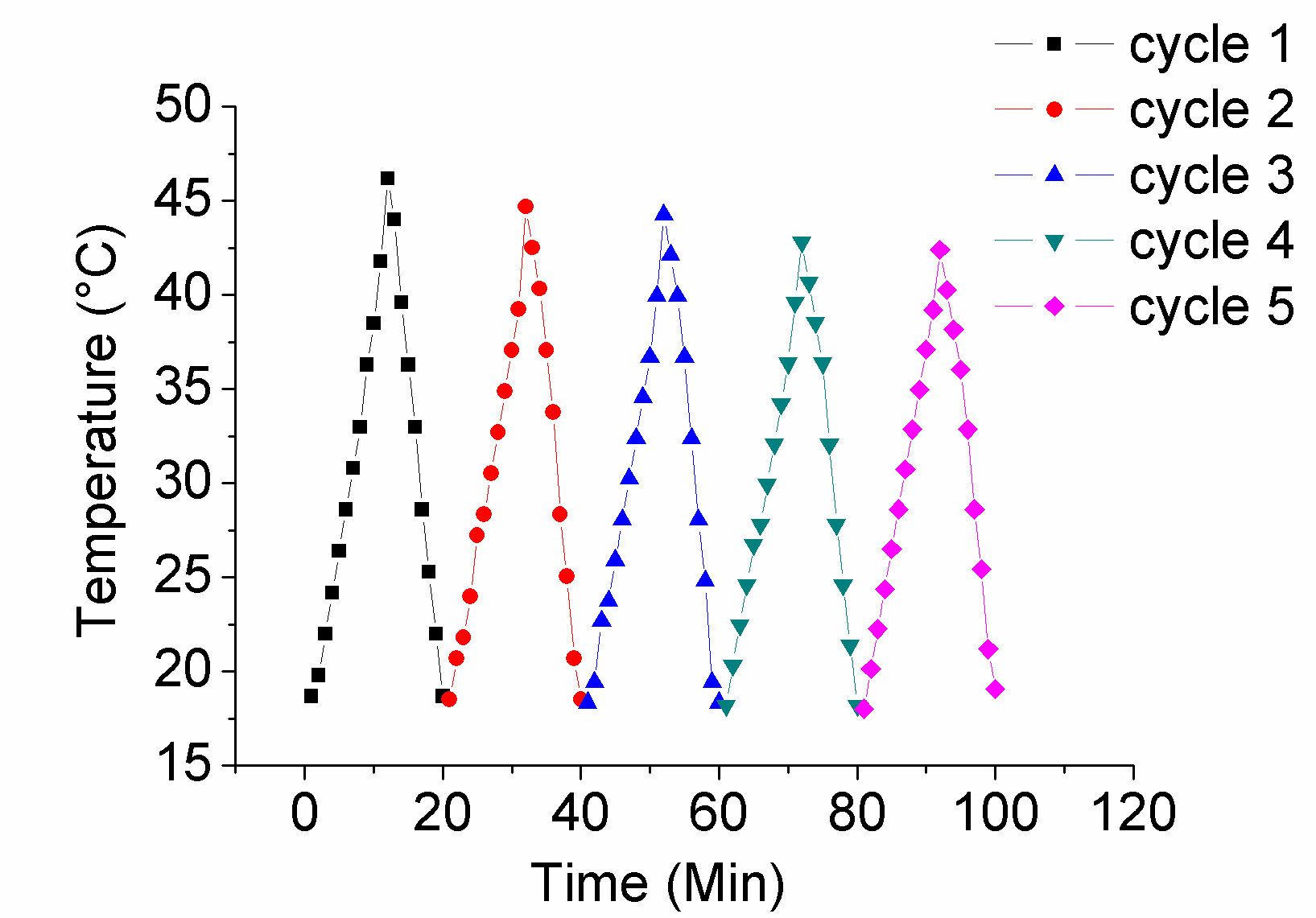
**

**Figure S3**. The photostability of the PDA NPs (2 mg/ml) after five cycles of NIR irradiation.


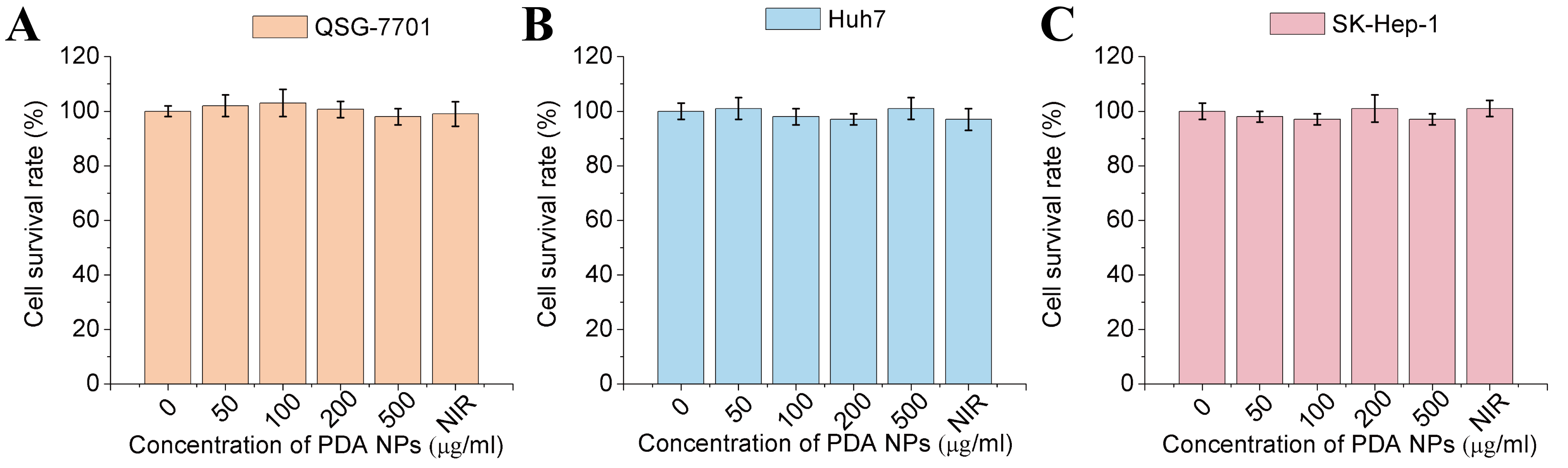


**Figure S4**. Biocompatibility of blank PDA NPs. Cell survival rate of (A) liver normal QGY-7701, liver cancer (B) Huh7 and (C) SK-Hep-1 cells after treating with different amounts of PDA NPs. Error bars represent mean ± SD (standard deviation, n = 3).

**
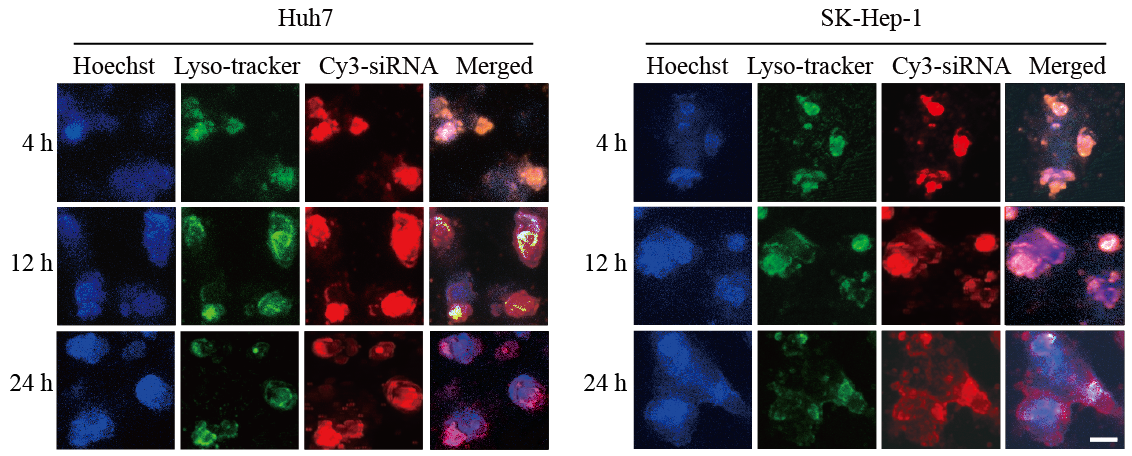
**

**Figure S5**. Fluorescence microscopy images of lysosomal localization experiment of the genetic nanomedicine. Liver cancer Huh7 and SK-Hep-1 cells incubated with lyso-tracker green for 1 hours after incubation with FA-modified Cy3-siRNA-loaded PDA nanomedicine for 4, 12 and 24 hours. The blue color is Hoechst, the green color is Lyso-tracker, the red color is Cy3-siRNA and the scale bar is 10 μm.

**
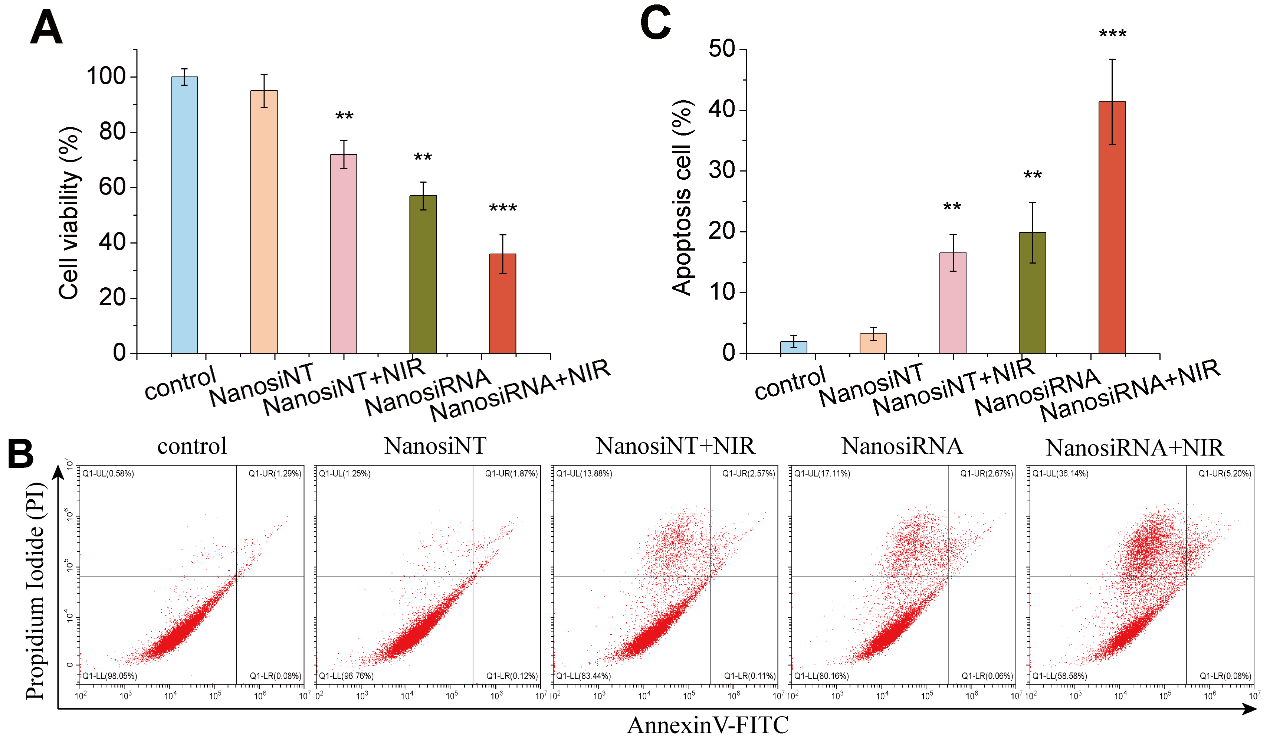
**

**Figure S6**. *In vitro* anticancer activity of the genetic nanomedicine combined with photothermal therapy in SK-Hep-1 liver cancer cells. (A) Viability of the SK-Hep-1 cells after incubation with siNT or siRNA-loaded PDA nanomedicine (containing 2 μg/ml of siRNA or siNT) in the absence or presence of laser irradiation for 48 hours. (B) Flow cytometric analysis of Huh7 cell apoptosis on the basis of Annexin V and FITC-PI staining. (C) Statistical analysis of apoptotic cells corresponding to (B). Error bars represent the mean ± SD (standard deviation, n = 3), **p< 0.01, ***p< 0.001.


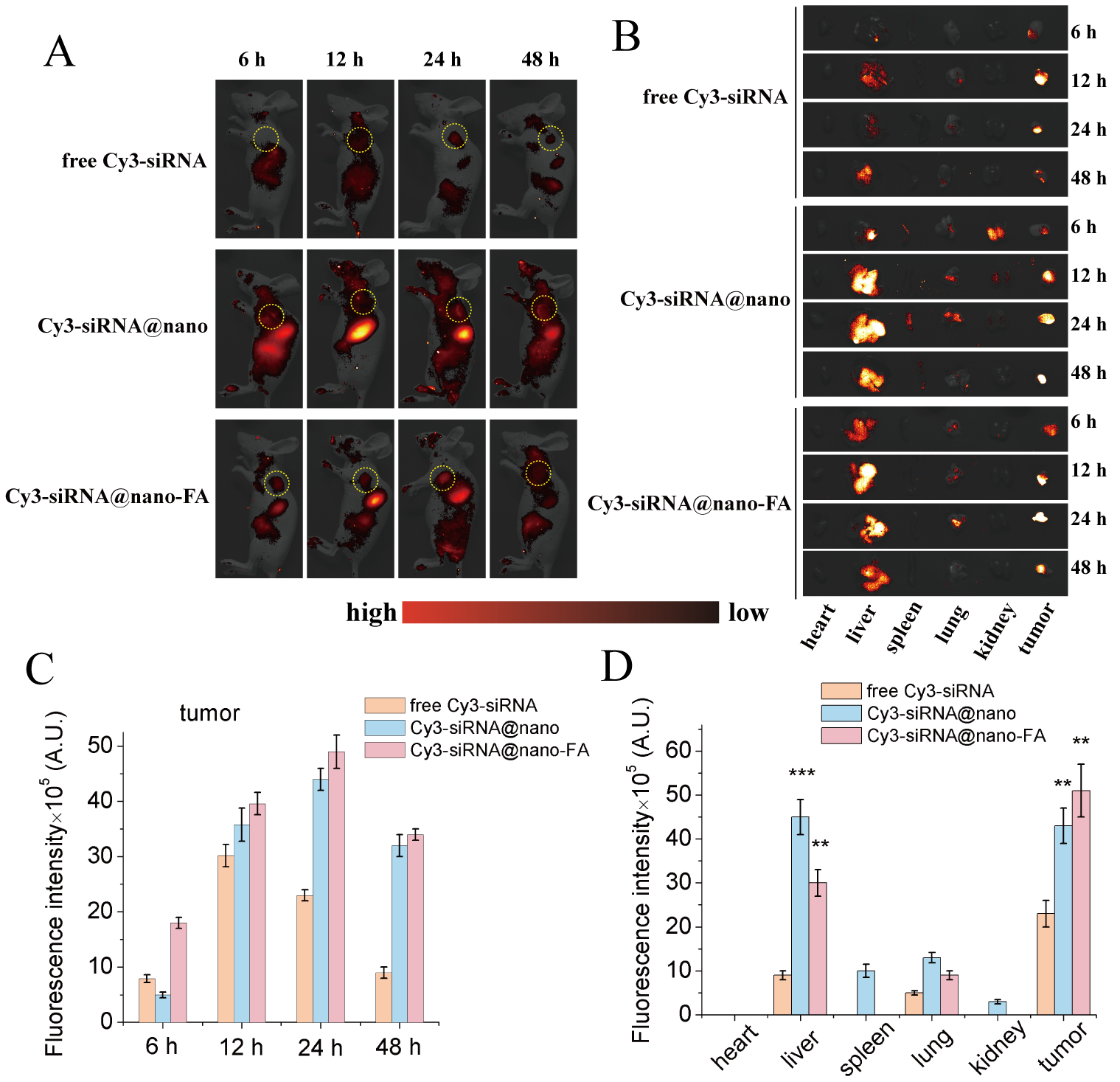


**Figure S7**. *In vivo* image of mice was observed at different time interval post-injection of free Cy3-siRNA, Cy3-siRNA loaded PDA nanomedicine, and Cy3-siRNA loaded FA-modified PDA nanomedicine. (A) Images of the aforementioned samples accumulation in tumors in live animals at different time interval. (B) *Ex vivo* fluorescence images of tumors and major organs (heart, liver, spleen, lung, kidney) at different time interval. (C) Fluorescence signals of Cy3 in tumors were quantified at different time interval. (D) Fluorescence signals of Cy3 in tumors and major organs were quantified at 24 hours, error bars represent the mean ± SD (standard deviation, n = 3), **p< 0.01, ***p< 0.001.


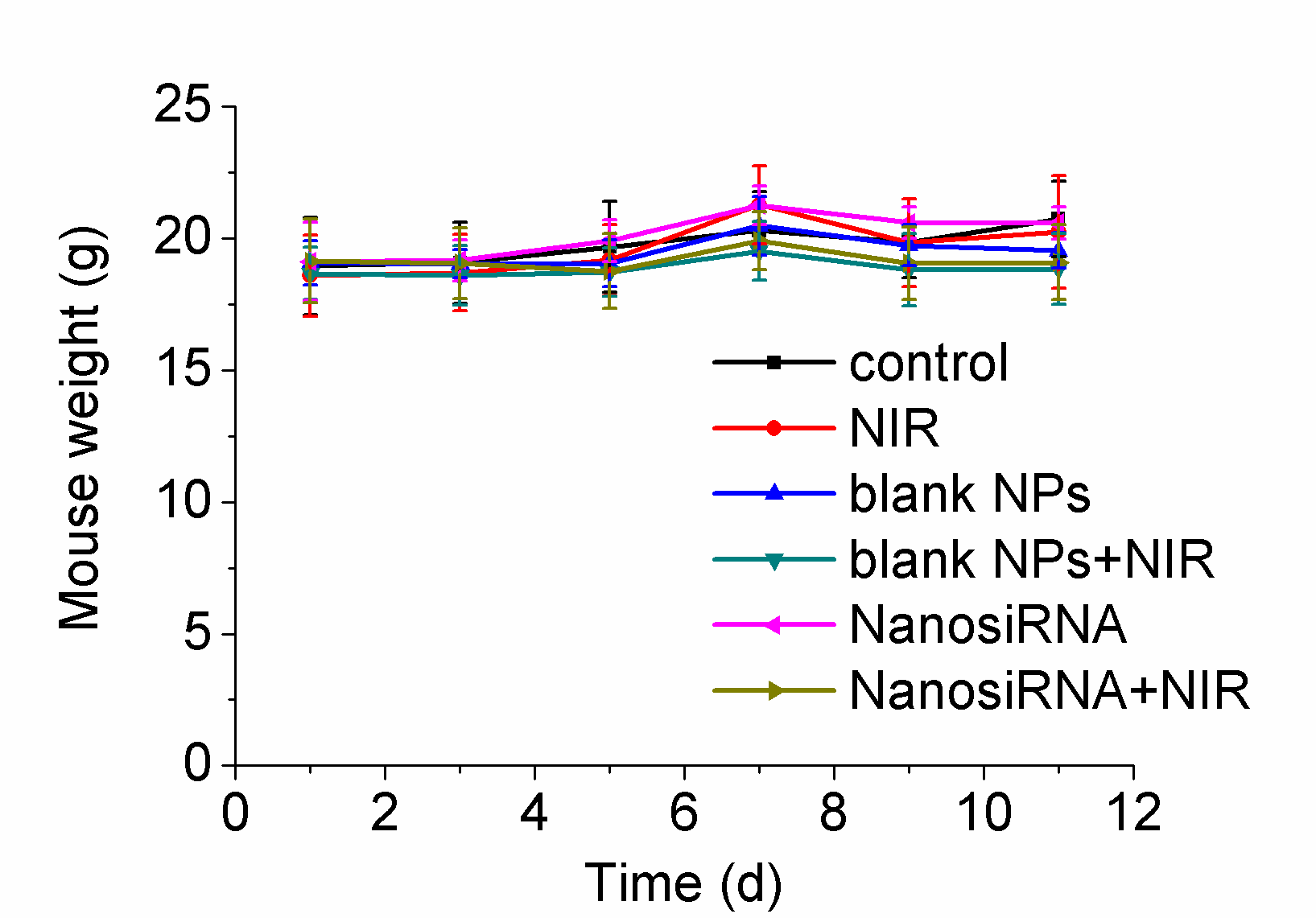


**Figure S8**. Mouse weight of Huh7 tumor after systemic administration of saline only, NIR laser irradiation, blank PDA NPs, blank NPs with laser irradiation, siRNA-loaded nanomedicine and siRNA-loaded nanomedicine with laser irradiation, error bars represent mean ± SD (standard deviation, n = 6).

**
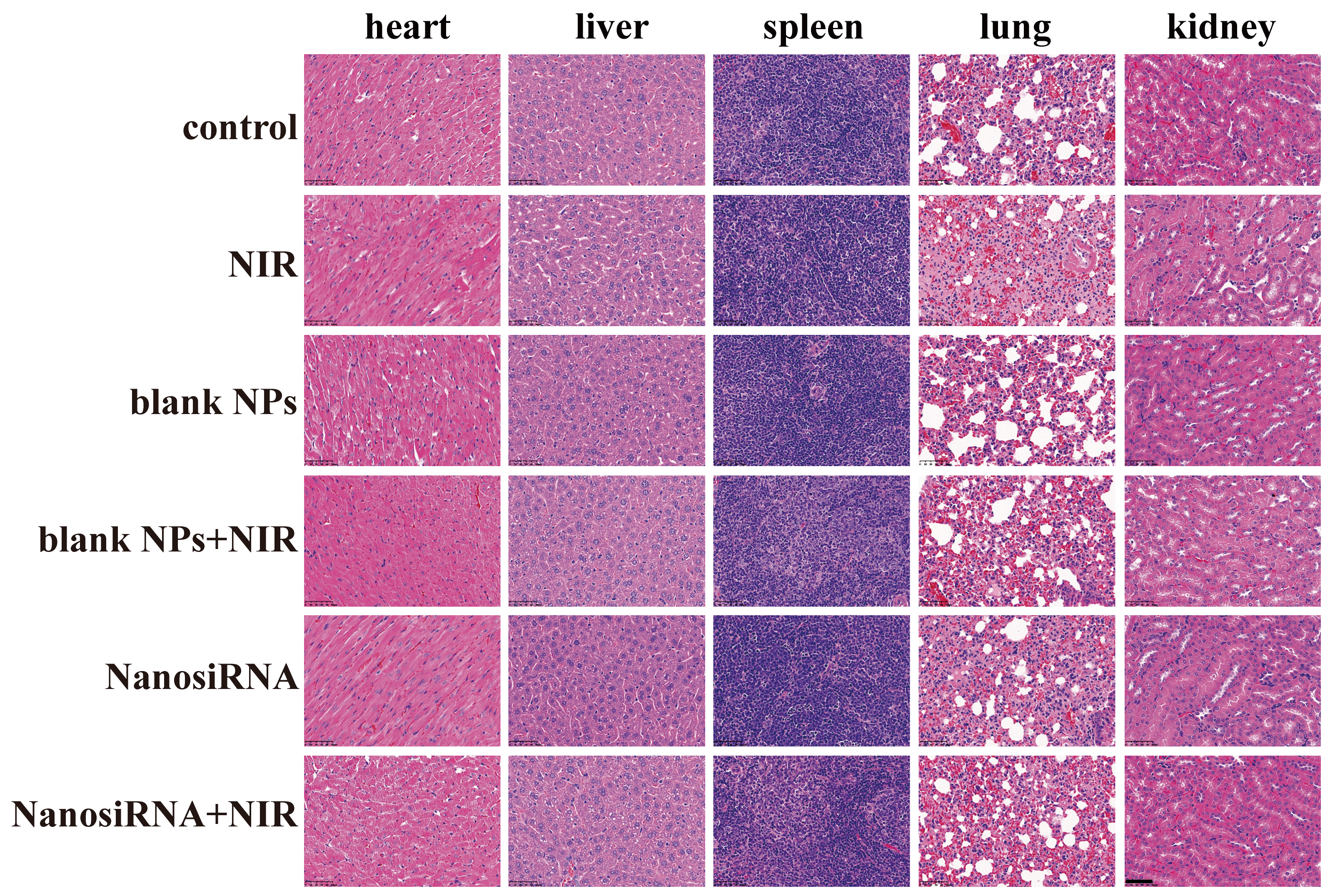
**

**Figure S9**. H&E staining images of major organs including heart, liver, spleen, lung, and kidney after the Huh7 tumor-bearing mice systemic administration with saline only, NIR laser irradiation, blank PDA NPs, blank NPs with laser irradiation, siRNA-loaded nanomedicine and siRNA-loaded nanomedicine with laser irradiation. Scale bar: 50 µm.
